# Use of WhatsApp in Children’s Oral Health Education Among Mothers in a Maternal and Child Cohort: Qualitative Content Analysis

**DOI:** 10.2196/89204

**Published:** 2026-10-15

**Authors:** Natália Gardin Varano, Ruann Oswaldo Carvalho da Silva, Julia Schlichting Azevedo, Juliana Schaia Rocha, Luciana Reis Azevedo Alanis, Deborah Carvalho, Renata Iani Werneck

**Affiliations:** 1 School of Medicine and Life Sciences, Pontifícia Universidade Católica do Paraná, Rua Imaculada Conceição, 1155, Prado Velho, Curitiba, Paraná, 80215-901, Brazil, +55 (41) 3271-2688; 2 Graduate Program in Information Management, Universidade Federal do Paraná, Curitiba, Paraná, Brazil

**Keywords:** health literacy, HL, health education, digital health, social media, WhatsApp, mobile health, mHealth, oral health, oral health literacy, child health, maternal and child health.

## Abstract

**Background:**

Research indicates that social media platforms play an important role in disseminating health information. Mothers, in particular, frequently seek guidance on child health through digital resources. Given the growing demand for health-related content, it is necessary to understand how these tools contribute to maternal health education, especially regarding the ability to interpret and apply health information.

**Objective:**

This study aims to investigate how WhatsApp influences the health education processes of mothers, as well as to identify the interests and competencies related to infant oral health among mothers participating in a maternal and child cohort.

**Methods:**

This was a qualitative, exploratory study grounded in the Bardin content analysis framework. Text messages exchanged between mothers and health professionals in a WhatsApp group linked to the Curitiba Maternal and Child Health Cohort (COOSMIC) were analyzed, referring to the years 2020 and 2021.

**Results:**

The corpus was composed of 36 dialogues: 28 concerning infant oral health and 8 regarding the group’s impact. The recording units were categorized by themes and interpreted according to emerging core meanings. We identified 2 main themes: eruption of primary teeth (mentioned 12 times) and bruxism in children (mentioned 5 times). Furthermore, in the topic related to the group’s impact, we observed a pattern among participants in recognizing the group as a source of support, guidance, and mutual help, highlighting the role of professionals and peer-to-peer exchanges in overcoming doubts and insecurities. Mothers demonstrated an active search for information and collaborative interaction with health professionals, establishing WhatsApp as a support and education network. Competencies linked to oral health literacy ranged from autonomy, empowerment, and security to insecurity, misinformation, and knowledge limitations regarding infant oral health information.

**Conclusions:**

WhatsApp proved to be a relevant tool for accessing information and guidance on infant oral health, favoring mutual support and communication among peers and professionals. The competencies and limitations identified in the mothers’ oral health literacy highlight the importance of professionally mediated educational strategies aimed at improving the quality of information and promoting autonomy in infant care.

## Introduction

Oral health constitutes an essential dimension of general health, as the oral cavity is directly related to quality of life [1]. From a broader perspective, health involves an individual’s living conditions and interactions with the environment, which are related to various social determinants [2]. In this context, education plays a prominent role, as it promotes the development of skills and competencies that contribute to quality of life. Thus, it is considered a prerequisite for health [3].

Among these skills, health literacy (HL) stands out, referring to the capacity to interpret, comprehend, and apply health information [4,5]. When this concept is applied to child health, maternal HL becomes especially relevant, since mothers play a fundamental role in the life and health of their children, directly influencing the formation of good habits [1]. Evidence indicates that children of mothers with higher oral HL have lower rates of dental caries [6], while low levels of oral HL negatively impact children’s oral health [7].

Research indicates that social media can play an important role in the dissemination of health information [8]. The active search for health-related content through digital resources has frequently been used by mothers interested in obtaining guidance on child care [9]. Furthermore, these resources offer connection spaces [10] that are specifically present in the maternal routine, providing emotional support and promoting the exchange of experiences between mothers [11,12].

With the growing use of social media by mothers seeking information about their children’s health, it becomes necessary to understand how these digital tools contribute to health education, especially concerning the maternal capacity to interpret and apply reliable information. Therefore, this study aims to investigate how WhatsApp (Meta Platforms, Inc) influences the health education processes of mothers, focusing on child health. Furthermore, it seeks to characterize interests in infant oral health, analyze the questions raised, and identify competencies related to infant oral HL within this population.

## Methods

### Study Design

This study is nested within a cohort of pregnant women, mothers, and their children included in the Curitiba Maternal and Child Health Cohort (COOSMIC), conducted by the Pontifical Catholic University of Paraná (PUCPR), in partnership with the Municipal Health Secretariat of Curitiba [13]. The cohort population includes pregnant women at any gestational stage, aged 16 years or older, residing in Curitiba and receiving care through the “Rede Mãe Curitibana” health network. Participants are followed from pregnancy until the child reaches 2 years of age. The cohort sample consists of 1046 participants, with a mean age of 27.95 years.

This is a qualitative, exploratory study design based on the analysis of secondary data derived from text messages within a WhatsApp group. WhatsApp is a real-time messaging platform that allows for text-based communication and the exchange of various media formats, including photos, videos, documents, and location data [14]. This group was created by the cohort to provide maternal and child health guidance to participants, with an average of 85 members, including mothers and the health care professional (HP) team.

### Data Source and Selection Criteria

Data were collected by exporting the WhatsApp group conversations to a text file, which exclusively contained text-based interactions between participating mothers and HPs. The selection of messages was conducted through manual reading, allowing for a comprehensive evaluation of the content shared in the group and ensuring that no relevant themes were missed. Additionally, the search function for specific terms (eg, tooth, mouth, and pain) within the text file was used to complement the manual reading. Only messages addressing doubts related to infant oral health were selected. Messages that did not specifically address this theme were excluded, ensuring that the corpus remained focused on the research’s central topic. In addition to this criterion, only data referring to the years 2020 and 2021 were analyzed.

### Data Analysis

Data interpretation was conducted using the content analysis method, as proposed by Bardin [15]. Through an exhaustive reading of the material, core meanings related to the investigated topic were identified. Initially, all dialogues were organized in a spreadsheet, where each row contained a question and its corresponding responses, preserving the original interaction flow. The material was reviewed repeatedly to identify recurring patterns in the mothers’ questions. Based on these patterns, dialogues addressing similar themes were grouped, allowing thematic categories to emerge inductively from the data. These categories subsequently served as the coding framework for data organization. The final count of dialogues was performed manually after the complete tabulation of all interactions. The coding and thematic categorization process was performed by the primary researcher (NGV) and subsequently reviewed and validated by a senior supervisor (RIW). Any disagreements or uncertainties regarding the definitions of the categories were resolved through consensus meetings between the researcher and the supervisor.

Data collected from the WhatsApp group were organized based on the doubts presented by the mothers. Each dialogue was defined as a single doubt (question), accompanied by one or more responses or interactions from other participants and HPs. Accordingly, dialogues were counted based on each new doubt sent to the group. A single mother could initiate more than one dialogue throughout the analyzed period; thus, the individual participant did not determine the number of dialogues. Cases where a question received no response were also recorded as initiated dialogues, even without subsequent interaction. In addition to dialogues related to infant oral health, a second thematic axis regarding “valuing the group as a support space” was analyzed. In these instances, dialogues were defined by spontaneous messages containing feedback from mothers about their experience in the group.

To facilitate visualization, consecutive messages sent by the same participant within a single dialogue were transcribed on a single line, separated by a forward slash (/). The dialogues were initially analyzed in Portuguese, maintaining the original spelling and informal language to preserve the authenticity of the interactions. For the purposes of this publication, the dialogues were translated into English. This process involved linguistic adaptations designed to ensure clarity and comprehension for the audience while carefully maintaining the informal tone and original intent of the participants’ messages.

For the analysis, a standardized nomenclature was adopted to identify participants. Mothers who initiated dialogues with questions or doubts were identified as “Mother A,” while those who participated in the interactions by responding were identified as “Mother B.” Responses from the HPs linked to the project were identified as “HP”. In addition to this classification, each participant was assigned a sequential number corresponding to the chronological order of their first intervention in the group. This numerical identification was maintained consistently throughout the entire analysis, regardless of whether the participant initiated new dialogues or interacted in dialogues started by others.

The use of this nomenclature and sequential numbering was adopted to preserve the identity of the participants, protecting personal information such as names and phone numbers. Beyond ensuring data confidentiality, this strategy facilitated the organization and visualization of the dialogues during the analytical process. All identifiers were removed, and quotes were deidentified before analysis to ensure the protection of participant information.

### Ethical Considerations

The COOSMIC project was submitted to and approved by the Research Ethics Committees of the PUCPR (approval numbers 4.819.028 and 2.672.385), and the Municipal Health Secretariat of Curitiba (approval number 4.859.861). The current analysis used secondary data from the COOSMIC cohort. At the time of inclusion in the cohort, participants provided informed consent authorizing the collection, storage, and future use of the data for research purposes. All data extracted from the WhatsApp interactions were completely anonymized before analysis and were used with authorization from the COOSMIC coordination team.

## Results

### Principal Findings

In the coding stage for the theme “infant oral health,” conducted in a Microsoft Excel spreadsheet, the selected material was reorganized and categorized, giving rise to the following themes: eruption of primary teeth, medication for children, bruxism in children, children’s oral hygiene, pacifier use in childhood, children’s products related to oral health, and questions about children’s oral health. Additionally, in a second spreadsheet, dialogues referring to valuing the group as a support space were arranged, in which spontaneous reports from participants praising the team and the group’s operation were observed. After defining the analysis corpus, the data were organized in a Microsoft Excel spreadsheet, totaling 36 dialogues: 28 related to oral health and 8 to the group’s impact.

The most recurrent themes related to “infant oral health” were eruption of primary teeth (mentioned 12 times) and bruxism in children (mentioned 5 times). Additionally, “valuing the group as a support space” was mentioned 8 times. The frequency of mentions of each theme by the participants is represented in Figure 1.

**Figure 1.**
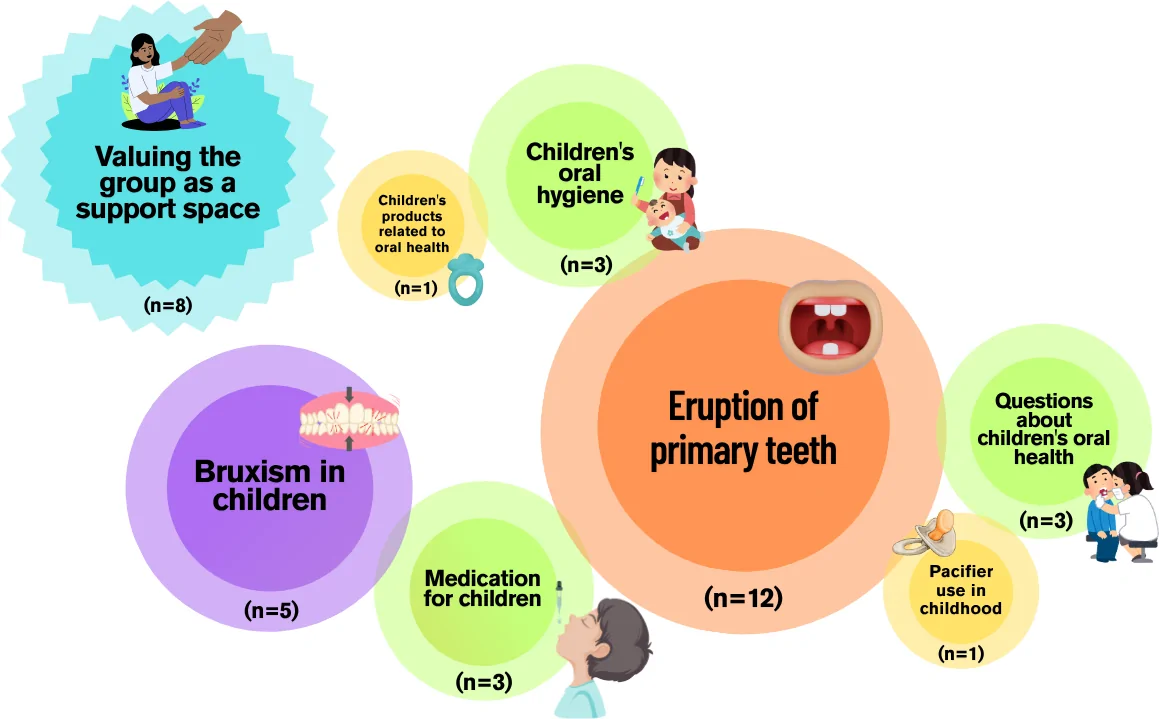
Frequency of themes related to infant oral health. Representation of themes, where the size and color of the circles vary according to the frequency with which each theme was mentioned in the dialogues. Larger circles indicate higher frequency of the theme. Colors correspond to the number of mentions: orange (n=12), blue (n=8), purple (n=5), green (n=3), and yellow (n=1). The number in parentheses (n) indicates the total number of times each theme was cited. The circle with serrated edges represents a theme not directly related to infant oral health and is shown in a distinct format, unlike the other circles, which represent health-related content.

From these data, it was possible to identify the most recurrent themes in the dialogues, as well as the frequency with which they were mentioned by the participants.

### Analysis of the Identified Categories

Based on this information, the second stage of the research proceeded, aiming to deeply comprehend the core meanings generated by the mothers regarding the themes discussed.

#### Eruption of Primary Teeth

##### Dialogue 1

***Mother A1:** Any dentist in the group? My daughter is very irritated with her teeth coming in... Besides Camomine [supplement used to relieve teething symptoms] and Nenedent [oral gel for gum pain and itching], what else can I do to relieve it?*

***HP:** You can use Nenedent up to 3 times a day... A teether or something soft, without sharp edges, for her to bite... Another tip is to gently massage...*

The mother’s interest in seeking further guidance and her appreciation for professional knowledge are evident. This behavior reflects an active effort to seek information about her child’s health in order to expand her knowledge and clarify her doubts.

##### Dialogue 2

***Mother A2:** Is anyone’s baby having teeth come in?*

***HP:** ...An alternative for relief is teethers with gel or water inside... you put it in the refrigerator to chill...*

***Mother B1:** My daughter has one with water... Very good...*

Mother B1’s response enriches the dialogue by validating the professional recommendation with her personal experience, illustrating an interaction between mothers and a HP.

##### Dialogue 3

***Mother A3:** My baby’s tooth came out 3 days before he turned 5 months 😱 My first child’s came out at 6 months. These children are too early*.

***Mother B2:** 😱😱😱😱*

***Mother B3:*** [*Name suppressed]’s first tooth came out late at 10 months...*

Beyond resolving doubts, the group functions as a peer support network for mothers, constituting a space where participants share experiences and forge social bonds.

##### Dialogue 4

***Mother A4:** How long does it take for a baby’s tooth to fully come out? It’s been a week that my daughter’s has only been tips!*

***HP:** That’s normal. The speed varies from child to child. If it has already broken through the gum, it will come in fully soon. Keep monitoring it*.

***Mother B4:** It took a month to be complete... These ones have been two weeks and are coming in very slowly*.

***Mother B5:** It’s normal, my 11-month-old baby’s is like that too. It takes weeks to come out completely*.

Mother A4’s inquiry is addressed with professional guidance regarding individual variations in tooth eruption timing, emphasizing the importance of continuous maternal monitoring. Furthermore, Mothers B4 and B5 contribute by sharing personal accounts of their children’s dental development, illustrating the exchange of experiences within the group.

##### Dialogue 5

***Mother A5:** My 9-month-old daughter has a fever and is very irritable. Both bottom teeth have come in and I can’t identify what it could be... should I take her to the emergency room?*

***Mother B6:** Take her to the doctor*.

***Mother B7:*** *Have the top ones come in? If not, it could be that too*.

In this interaction, Mother B7’s intervention stands out as she hypothesizes that the child’s irritability and discomfort may be associated with the eruption of the upper teeth.

### Medication for Children

#### Dialogue 6

***Mother A6:** Girls, does anyone here give iron [supplement used to prevent anemia] to their baby? The pediatrician prescribed it, but I see that some children develop rotten teeth when given it. Is it mandatory to give?*

***Mother B8:*** *I give it every day. I haven’t seen this “rotten tooth” thing, but it was recommended to avoid anemia. I also give Adtil [vitamin A and D supplement]. You have to clean the baby’s mouth after giving it, to avoid cavities, according to the baby’s dentist*.

***Mother B9:** I give it. And I haven’t had any problems with cavities*.

***Mother B10:** I give it and I also give vit. [vitamins] Do I clean the mouth after giving the iron ?*

***HP:** When pediatricians prescribe vitamins (like Ad-til) or minerals (like iron), these serve to supplement the body’s nutrient amounts and should be given. Ferrous sulfate can stain teeth.... That’s why cleaning the mouth after administering prevents these stains*.

Mother A6 shares her inquiry regarding the necessity of iron supplementation and her concerns about potential dental damage, while Mother B8 recounts her experience and the hygiene instructions provided by a dentist. The dialogue features an intervention by a HP, who clarifies the importance of supplementation and provides guidance on oral hygiene practices to prevent dental staining.

#### Dialogue 7

***Mother A7:** ...My son’s first tooth is showing, so I bought Nenedent [oral gel for gum pain and itching] ointment and applied it for the first time today. I would like to know how often I can use it? How many times a day? 😊... Can it be close to mealtimes?*

***HP:** ...every 6 hours... It can be after, because then the gel stays in contact with the gum longer...*

In this dialogue, Mother A7 seeks detailed instructions regarding the frequency of use and the appropriate timing for applying an oral gel to her child.

#### Dialogue 8

***Mother A8:** ...[Name suppressed] is 5 months old and his first tooth broke through the gum today. He’s in pain, and very tearful. Can I give him baby paracetamol [acetaminophen]?*

***Mother B11:** My experience with my [name suppressed] only the original Camomilina C [herbal medicine used to relieve teething symptoms] worked... even generic didn’t help🤦🤷*

***HP:** Yes, you can give Tylenol baby [acetaminophen formulated for infants].... Draw up the amount corresponding to his weight in the syringe and... every 8 hours... You can give it for 5 days*.

***Mother A8:** I gave paracetamol [acetaminophen], 8 drops directly into his mouth. I did it wrong. Did it really have to be in the syringe??*

In this scenario, Mother A8 seeks guidance regarding the possibility of administering medication to her child and subsequently expresses doubts about the correct method of administration, even after receiving instructions from the HP.

### Bruxism in Children

#### Grouped Dialogues

Similar dialogues were identified among Mothers A9, A10, and A11, who expressed doubts related to their children’s habit of grinding their teeth. It was observed that Mothers A9 and A11 received guidance from HPs, while Mother A10 received an answer from another group participant:

***Mother A9:** I have a question about my son, is it normal for him to grind his teeth? He is almost 12 months old*.

***HP:** It is very common at this age. Take a look at this image I sent!*

***Mother A10:** My 7-month-old baby is having teeth come out. But she keeps grinding her teeth, it gives me anxiety 😊🤔*

***Mother B12:** My son used to “weirdly move” his jaw... it gave me anxiety..lol I used to tell him “Stop that.” But he stopped on his own, never did it again. Oops: “Clicking”*

***Mother A11:** My girl is 8 months old and has 4 teeth and grinds her teeth, is it normal? She grinds all the time, it seems like they will break*

***HP:*** *It is very common at this age. Only if the teeth are wearing down too much is a dentist’s examination necessary. But up to 2 years of age, the behavior is normal. I sent an image for you to read more about this habit*

In these interactions, mothers share similar experiences and concerns regarding tooth grinding (bruxism) in their children. Consequently, the HP intervenes by providing technical guidance and informative images aimed at addressing the doubts and concerns expressed by the participants.

#### Dialogue 9

***Mother A12:** Guys, my baby started grinding his teeth 😱😱😱 I keep trying to make him stop. He is 10 months old... He grinds during the day. Not at night*

***HP:** This habit is very common at his age. It is only worrying when he hurts himself or his teeth wear down significantly. I will forward an image with more explanations*

***Mother B13:*** *I have bruxism... I have 4 broken teeth... I had several problems, the best thing is early diagnosis and treatment*

In this dialogue, a similar situation is observed with Mother A12, who expresses concern regarding her child’s habit of bruxism. In response, the HP provides guidance and seeks to reassure her.

### Children’s Oral Hygiene

#### Dialogue 10

***Mother A13:** ...Is cleaning the baby’s mouth after every feeding and meal? I’m doing it after meals... The teeth haven’t come in yet, but it’s very swollen and almost coming out. I’m cleaning with a cloth and water, I don’t know if that’s correct. She is seven months old*

***Mother B14:** It’s recommended to clean with gauze 3 times a day. I only clean when bathing 🥺🙈 when I give iron [supplement to prevent anemia], after a few minutes. Just so she doesn’t get thrush on her tongue. But if there is enough time, you can clean more often*

Mother A13 demonstrates attentiveness to her daughter’s oral hygiene by seeking validation of her existing routine. In addition, Mother B14 provides detailed information regarding frequency, materials, and the underlying purposes of the practice, sharing guidance that reflects previous access to technical knowledge on infant oral health.

### Pacifier Use in Childhood

#### Dialogue 11

***Mother A14:** ...My daughter uses a pacifier, and she turned 6 months old. Do I need to change her pacifier to the 6 to 12 month pacifier?*

***Mother B15:** I changed it. My son uses the Mam brand. He used number 1 until six months, then I changed. He accepted it very well. Because Mam pacifiers are all anatomical and always follow the same model, only the size changes... Everything has to evolve as the child evolves... That’s why everything is right according to the child’s age, right?*

***Mother B16:** In the beginning, it’s strange because of the size... But then you’ll see that it fits perfectly.. lol*

In this interaction, Mother B15 provides a detailed personal account of the transition to a new pacifier size and her child’s adaptation to the product. Mother B16 complements the dialogue by reporting her own perceptions and initial experiences regarding the size change, thereby illustrating the peer interaction and exchange of experiences among mothers within the group.

### Children’s Products Related to Oral Health

#### Dialogue 12

***Mother A15:*** *What kind of teether should I buy?*

***Mother B17:*** *Here I have that little hand from BDA, a butterfly, and a Fisher Price one... There was never a risk of her biting my chest due to confusion. Don’t buy a very soft one so there’s no confusion during breastfeeding*

***HP:** ...those with gel or water inside are great! When the teeth are coming in, you can put them in the refrigerator... The cold relieves local irritation*

In this dialogue, Mother A15 receives information from distinct sources. While Mother B17 shares a personal account, the HP provides guidance based on technical knowledge. This scenario highlights the diversity of information circulating within this digital environment.

### Questions About Children’s Oral Health

#### Dialogue 13

***Mother A16:*** *My baby has a black spot on his tooth... I don’t know what it is. It looks painted, you know? Can anyone tell me if it’s normal?...*

***HP:*** *Is your child taking any iron medication? We can talk better in private contact*.

***Mother A16:*** *He takes ferrous sulfate. You can call me*

Mother A16 demonstrates attentiveness and vigilance regarding her child’s oral health by reporting observed changes and seeking guidance. In response, the HP seeks to understand the clinical condition in order to provide appropriate guidance. This interaction illustrates how this digital environment facilitates direct communication between the HP and the participant.

### Unanswered Questions

During the analysis, doubts raised by participants related to infant oral health were observed; however, these did not have continuity in the dialogue, whether due to a lack of response or a lack of interaction. These records nevertheless highlight mothers’ active and recurrent efforts to seek information related to their children’s oral health:

***Mother A17:*** *My[name suppressed] is 5 months old, complaining about gum itching and certainly pain too. I’ve used Camomine C [supplement used to relieve teething symptoms], Nenedent [oral gel for gum pain and itching], Ibuprofen is helping a little... Do you have anything to suggest that helps minimize the discomfort? Please share with me if you have anything to recommend*.

***Mother A18:*** *I have a question, do I clean after her teeth start to come in or even before?*

***Mother A19:*** *My 5-month-old baby’s teeth are coming in, he is very tearful and not sleeping well. What can I do?*

***Mother A20:*** *Any tips for when the first tooth breaks through?*

***Mother A21:*** *How do I perform good oral hygiene if my baby no longer opens his mouth or bites?*

***Mother A22:*** *Guys, my baby will be 2 months old and doesn’t take her hand out of her mouth, she’s very irritable 🥺*

The excerpt below reveals a series of important elements related to the barriers faced by mothers during the COVID-19 pandemic:

***Mother A23:*** *...I believe the back teeth are coming in (I know because I managed to see, and the gum is super swollen), but she puts her hand in her mouth a lot and complains. Would anyone be able to help me with what to do? Keeping in mind that we are in the COVID-19 pandemic and taking her to the dentist at this moment is complicated. Not to mention that she is autistic, and she has certain rejections*.

The situation described by the mother highlights the difficulties faced in accessing in-person dental care, especially when caring for children with special needs during the health crisis. In this scenario, the group was configured as an accessible support tool given the limitations imposed by the pandemic.

### Audiovisual Questions

#### Dialogue 14

During the analysis, dialogues were observed in which participating mothers used audiovisual media to express their doubts, such as audios, photographs, and videos. However, access to the WhatsApp group interactions was through a text file, making it impossible to view and analyze these other media formats. Therefore, only textual messages were considered. To signal the use of these media, brackets were inserted to indicate the type of media shared by the participant:

**Mother A24:** *[sent image] It’s purple, looks like a sore. He had a fever of almost 40 degrees. The COVID test was negative*.

***Mother B18:*** *That tooth is the most painful one, poor thing. There is Nenedent [oral gel for gum pain and itching]... when my children had this discomfort, I applied the ointment and alternated with Novalgin infant solution [dipyrone oral solution for infants]. And I also gave those cold teethers to bite. Check correctly with the pediatrician or pharmacist how much Novalgin you can give, to help with the pain he is feeling*.

***HP:** You can use Nenedent or Ad Muc gel [both topical oral gels used to relieve gum discomfort during teething]. Apply about 4 times a day. And you can use an analgesic to help too. The most suitable for babies is Paracetamol [acetaminophen]. 1 drop per kilogram of weight every 8 hours*.

***Mother B19:*** *[link suppressed] Watch this video. Cold things help a lot*

***Mother B20:*** *Mallow tea or mallow compress helps a lot*

***Mother B21:*** *How many months is he? My baby is also 8 and doesn’t have any teeth, I’m starting to worry*

***Mother B22:*** *...teeth can take up to 1 year of age to come in. My friend’s son had his first ones come in when he turned 1 year old*.

Following Mother A24’s question, several interactions took place among the participants, including guidance, suggestions, and the sharing of personal experiences. Mother B19’s sharing of a web link illustrates mothers’ use of social media to seek health-related information.

#### Dialogue 15

***Mother A25:*** *[sent audio and image, indicated by the responses as a question regarding the eruption of the baby’s dentition]*

***Mother B23:*** *Oh my God. Mine will turn one and it’s swollen and still no sign of the first one 🤦🏻*

***Mother B24:*** *...I think it’s not a rule... Mine’s first ones came out at 7 months, one on top and one on the bottom... Then the others came out*.

***Mother B25:*** *Mine had two bottom teeth come out between 5 and 6 months. Now the top is mega swollen for 1 month already and hasn’t come out... He turned 10 months*

Based on the audio message and an image shared by Mother A25, the other participants contributed by sharing their personal experiences regarding the timing and sequence of their children’s tooth eruption. This type of interaction illustrates the exchange of similar experiences among mothers.

#### Dialogue 16

**Mother A26:** *[sent audio, indicated by the responses as a question regarding bruxism in children]*

***Mother B26:** It is normal for the baby to grind his teeth. Also because he is discovering himself and because he doesn’t have a posterior bite.... But regarding taking the baby to the dentist, this can be done from the first month, to learn how to care...*

In this interaction, Mother B26 responds to the audio message sent by Mother A26, reassuring her about bruxism in children. In addition, she highlights the importance of an early dental consultation, demonstrating confidence and clarity when sharing knowledge about children’s oral health.

#### Dialogue 17

***Mother A27:** [sent image] My mother said it’s thrush*.

***Mother B27:*** *Does it look like angular cheilitis? Or an aphthous ulcer? Is there only one? Did you notice if she feels pain there?*

***Mother B28:** Only one? Does she have a fever?*

***Mother B29:** Something she ate that was hot*.

***Mother B30:** Thrush. Put gentian violet on it. Put it on a gauze and apply it to the baby’s mouth*.

***HP:** It could be stomatitis*

In this dialogue, Mother A27’s question prompted a series of responses and hypotheses from the other participants, ranging from informal diagnostic suggestions to recommendations for home remedies, such as the use of gentian violet. This interaction highlights the high volume of information and the diversity of guidance shared within the digital environment.

#### Dialogue 18

***Mother A28:** [sent image] Hello, my daughter is 2 months old, and I found this white spot.... Could it be a tooth trying to come out?*

***HP:** This spot is likely what we call an Epstein pearl... It is just an alteration in the gum... but it disappears on its own*.

***Mother A28:** Thank you.... it’s not a tooth! Lol! 😉*

The intervention of the HP was essential in clarifying the mother’s concern, promoting her understanding of an unexpected clinical sign. By providing a clear explanation, the HP enabled the mother to correctly interpret the information received, thereby providing reassurance.

### Valuing the Group as a Support Space

Reports shared by the mothers reveal the importance attributed to the group as a support space during motherhood. Participants highlight the exchange of experiences and support as differentiators in the maternal experience:

***Mother A29:** Welcome[name suppressed], this group is great, we exchange a lot of experience and help each other too 👏🏻🥰🙏🏻😘*

***Mother A30:** This group is truly blessed 🙏🏻😘 We are blessed to have the support of professionals 24/7 here 🙏🏻*

***Mother A31:** Guys, the best thing about being a mother is having this group and this exchange of experiences we have here, it’s very good to be able to share our experiences*

***Mother A32:** ...It’s great when you are a first-time mother and have a group like this to clear up doubts...*

***Mother A33:** You are angels! The whole team! Thank you very much! 😘🙏🏼💚”*.

***Mother A34:** This team rocks ❣️ I am also very grateful to every professional present here... you are fundamental in our lives and our children’s lives!...*

***Mother A35:** Thank you[name suppressed]! Your call was very important. I felt great 🥰*

***Mother A36:** Just stopping by to congratulate this Coosmic team! ❤️🍀*

Finally, the mothers expressed their appreciation for and satisfaction with the group, highlighting positive aspects such as the opportunity to participate in this space, the possibility of clarifying doubts, the support received, and the strengthening of the support network. In addition, they expressed gratitude for the support provided by the HPs.

## Discussion

### Principal Findings

This study contributes to the understanding of health education delivered through WhatsApp, highlighting mothers’ proactive interest in seeking information about children’s oral health. Health guidance provided by HPs through the platform was associated with positive outcomes, facilitating the resolution of mothers’ questions while strengthening a peer support network. Our findings further reinforce the idea that the active involvement of HPs is essential to ensure the reliability of oral health information disseminated and shared through digital platforms.

The findings of this study reveal that mothers actively seek information related to their children’s oral health through WhatsApp. This behavior reflects their interest in expanding their knowledge and clarifying doubts, as observed in *Dialogue 1* and *Dialogue 11* and in the *Unanswered Questions*. These findings are consistent with the literature, which indicates a growing trend among mothers to use social media as a source of information on child health [9,11].

Beyond information seeking, interactions within the group revealed other relevant behaviors. Among these, the search for opinions, observed in *Dialogue 14*, stands out. Previous studies have also shown that mothers use digital platforms to support decision-making regarding childcare, attributing considerable value to these spaces as sources of information [11]. Another identified aspect was the exchange of experiences, advice, and suggestions among participants. This pattern, observed in *Dialogue 3*, *Dialogue 4*, *Dialogue 14*, and *Dialogue 15*, reinforces collective support and highlights a frequent need for connection and validation of motherhood experiences [11].

In this context, the group extends beyond its role as a space for clarifying doubts and functions as a peer support network, providing an environment in which participants share experiences, recognize common situations, and establish social bonds. This environment is valued by mothers for promoting mutual validation, reassurance, and support, as reported in the findings of this study. Similar findings have been reported in previous studies, which indicate that digital platforms can contribute to mothers’ emotional well-being [11] by fostering support, information sharing, and a stronger sense of connection among participants [12].

This study also demonstrates that WhatsApp can be used as a strategy for health promotion. Across several dialogues, HPs play a key role by providing guidance that promotes maternal autonomy. This is illustrated in *Dialogue 4*, in which the mother is advised to monitor her infant’s tooth eruption, and in *Dialogue 6* and the *Grouped Dialogues*, where HPs help clarify questions and improve participants’ understanding. These findings highlight the potential of social media as a strategic tool for health education [16]. Furthermore, they underscore the importance of professional guidance, as clear explanations enable mothers to correctly understand and independently apply health information, as illustrated in *Dialogue 18*.

In this context, WhatsApp facilitates communication between HPs and mothers. This practice has also been described in previous studies, in which HPs use digital technologies to provide health guidance and information [17,18], reflecting the field of teledentistry, which refers to the use of technologies, such as mobile communication devices, to deliver dental services and health education activities [19]. In *Dialogue 13*, the HP’s suggestion to continue the conversation privately illustrates the delivery of health education through remote care, characterizing a teledentistry practice. This approach has emerged as an alternative for providing health guidance while strengthening the relationship between HPs and patients [18]. Similarly, another study identified a similar scenario, highlighting teledentistry as a tool that can be used by HPs for oral health promotion, particularly through text messages and mobile apps [20].

Teledentistry gained particular prominence during the COVID-19 pandemic [21], when limited access to in-person dental care led digital technologies to become an accessible resource for addressing health-related concerns [8,18]. In the *Unanswered Questions* section, mothers sought guidance regarding their children’s oral health during this period, using the group as one of the available sources of professional support. This finding further reinforces the potential of WhatsApp as a tool for delivering health guidance and information.

However, these digital interactions also reveal potential risks and limitations. In *Dialogue 9*, 1 mother compares her experience of bruxism in adulthood with another mother’s report of bruxism in her child during infancy. This example illustrates the risk of sharing inaccurate information that is not supported by scientific evidence. This is particularly important because bruxism is considered a common finding in early childhood [22], whereas in adults it is associated with different etiologies [23].

Another example is observed in *Dialogue 17*, in which a mother recommends applying gentian violet to the infant’s mouth. Information shared by individuals without health care training may contribute to the phenomenon known as an infodemic, characterized by an overabundance of information, often including unreliable content, that can compromise health promotion [24]. A similar situation is observed in *Dialogue 12*, where a participant states that soft teething rings should not be used because they could interfere with breastfeeding. This recommendation contradicts current evidence supporting the use of soft, flexible teething rings because of their significant soothing effect during tooth eruption [25]. Therefore, unreliable information may circulate within the group, highlighting the importance of HPs in providing accurate, evidence-based guidance [24].

Taken together, these findings emphasize the importance of the presence and active mediation of HPs. Their involvement is essential to provide technical clarification, prevent the dissemination of inaccurate information, and promote access to reliable health information. Conversely, the absence of such active mediation may increase maternal uncertainty and contribute to the spread of misinformation [24].

Although inaccurate information was shared by some participants within the group, the findings also identified mothers who demonstrated knowledge related to children’s oral health. In *Dialogue 5*, for example, 1 mother critically assessed the situation by suggesting that tooth eruption could be the cause of her infant’s discomfort, demonstrating a higher level of oral HL. This ability reflects critical appraisal skills [4,5], as she associated a clinical sign—irritability, which is commonly related to the eruption of primary teeth [17]—with the timing of tooth eruption.

In *Dialogue 7* and *Dialogue 10*, mothers sought information regarding the appropriate use of medications and oral hygiene practices for their children, suggesting more advanced HL skills by demonstrating the ability to seek, understand, and apply health information—competencies typically associated with individuals who are better able to manage health-related situations, as described in the literature [5]. These HL skills were also evident in other situations, including mothers’ vigilance regarding their children’s oral health, as observed in *Dialogue 13*, and their confidence in discussing bruxism and recognizing the importance of early dental care, as illustrated in *Dialogue 16*.

Conversely, *Dialogue 8* illustrates HL skills that were still developing. The mother’s uncertainty regarding the correct administration of the medication, even after receiving guidance, suggests difficulties in understanding and applying health information in practice. This dialogue reflects competencies consistent with more basic levels of HL, in which health information is applied with less autonomy than in more advanced manifestations of HL [5].

Taken together, these findings indicate predominantly advanced manifestations of HL among the participating mothers, reflected in a more proactive attitude and a greater understanding of children’s oral health information. This familiarity with the topic was demonstrated not only through the ability to interpret health information but also through the ability to share it with others, thereby contributing to collective knowledge [5]. Furthermore, greater attention to children’s oral health care was observed, consistent with previous studies which show that higher levels of maternal HL are associated with increased attention to child health care [6,26].

### Limitations

This study presents some limitations. First, the number of text messages collected for the analysis of infant oral health is small, which restricts the analysis and may not reflect the diversity of experiences among mothers. Second, as this research is based exclusively on text messages, it was not possible to directly evaluate the impact of the information on mothers’ daily lives, which limited understanding of the effectiveness of digital technologies within health education processes. To address this, complementary data, such as interviews, observations, or objective outcome measures, would be necessary. Given these limitations, it is recommended that future studies use mixed methods, integrate impact measures, and include longitudinal evaluations, in order to deepen the understanding of the effectiveness of digital technologies in mothers’ health education processes.

### Conclusions

WhatsApp proved to be a useful tool for mothers seeking guidance related to infant oral health. Participants reported positive impacts resulting from the guidance received from HPs, as well as the peer support built within the group. Furthermore, the study allowed for the identification of a diverse range of patterns related to the competencies and limitations in infant oral HL within the studied population, such as empowerment, autonomy, insecurity, knowledge gaps, and misinformation.

## Supplementary material

10.2196/89204 Multimedia Appendix 1 Transcripts of ChatGPT conversations.

10.2196/89204 Multimedia Appendix 2 Transcripts of NotebookLM conversations.

## Abbreviations

COOSMIC: Curitiba Maternal and Child Health Cohort
HL: health literacy
HP: health care professional
PUCPR: Pontifical Catholic University of Paraná

## References

[1] (2018). A Saúde Bucal no Sistema único de Saúde [Report in Portuguese]. https://bvsms.saude.gov.br/bvs/publicacoes/saude_bucal_sistema_unico_saude.pdf.

[2] (1986). Conferência Nacional de Saúde: relatório final [Report in Portuguese]. https://bvsms.saude.gov.br/bvs/publicacoes/8_conferencia_nacional_saude_relatorio_final.pdf.

[3] (1986). Ottawa Charter for health promotion. https://iris.who.int/items/9e6f3c72-3669-4f82-a3e2-990b1c4df471.

[4] Lynn NB, Panzer AM, Kindig DA, Institute of Medicine (US) Committee on Health Literacy (2004). Health Literacy: A Prescription to End Confusion.

[5] Nutbeam D, Lloyd JE (2021). Understanding and responding to health literacy as a social determinant of health. Annu Rev Public Health.

[6] Marandi M, Babaei A, Momeni Z (2025). Association of maternal oral health literacy with dental caries status of 6-9-year-old children according to the caries assessment spectrum and treatment (CAST) index. BMC Oral Health.

[7] Kamolchaiwanich K, Lee JY, Leelataweewud P (2025). Exploring the link between maternal oral health literacy and child oral health behaviours. Int Dent J.

[8] Lima JR, Oliveira MCP, Lima KMO (2021). Health education strategies for pregnant and puerperal women in coping with the COVID-19 pandemic. Res Soc Dev.

[9] Hanach N, Saqan R, Radwan H, Baniissa W, de Vries N (2024). Perceived experiences and needs of digital resources among postpartum women in the United Arab Emirates: qualitative focus group study. J Med Internet Res.

[10] Fialho J (2015). Pressupostos para a construção de uma sociologia das redes sociais [Article in Portuguese]. Sociologia.

[11] Henton S, Swanson V (2023). A mixed-methods analysis of the role of online social support to promote psychological wellbeing in new mothers. Digit Health.

[12] Yamashita A, Isumi A, Fujiwara T (2022). Online peer support and well-being of mothers and children: systematic scoping review. J Epidemiol.

[13] Bettega PVC, Rocha JS, Carvalho DR (2022). Coorte de Saúde Materno-Infantil de Curitiba (COOSMIC): protocolo de estudo. R Saúde Públ Paraná.

[14] (2026). WhatsApp.

[15] Bardin L (1977). Análise de Conteúdo [Book in Portuguese].

[16] Taheri Z, Bakouei F, Delavar MA, Faramarzi M, Bakhtiari A, Amiri FN (2022). Effectiveness of distance education program on mothers’ empowerment in exclusive breastfeeding: a randomized clinical trial. J Educ Health Promot.

[17] Fitzpatrick PJ (2023). Improving health literacy using the power of digital communications to achieve better health outcomes for patients and practitioners. Front Digit Health.

[18] Giudice A, Barone S, Muraca D (2020). Can teledentistry improve the monitoring of patients during the COVID-19 dissemination? A descriptive pilot study. Int J Environ Res Public Health.

[19] (2023). Guide to understanding and documenting teledentistry events. https://www.ada.org/-/media/project/ada-organization/ada/ada-org/files/publications/cdt/v4_d9995andd9996__adaguidetounderstandinganddocumentingteledentistryevents_2023jan.pdf.

[20] Fernández CE, Maturana CA, Coloma SI, Carrasco-Labra A, Giacaman RA (2021). Teledentistry and mHealth for promotion and prevention of oral health: a systematic review and meta-analysis. J Dent Res.

[21] Howell SEI, Kumar P, da Costa J (2023). Teledentistry adoption and applications: an American Dental Association Clinical Evaluators Panel survey. J Am Dent Assoc.

[22] Storari M, Serri M, Aprile M, Denotti G, Viscuso D (2023). Bruxism in children: what do we know? Narrative review of the current evidence. Eur J Paediatr Dent.

[23] Vieira AR, Scariot R, Gerber JT (2020). Bruxism throughout the lifespan and variants in MMP2, MMP9 and COMT. J Pers Med.

[24] Briand S, Hess S, Nguyen T, Purnat TD, Purnat TD, Nguyen T, Briand S (2023). Managing Infodemics in the 21st Century: Addressing New Public Health Challenges in the Information Ecosystem.

[25] Lerond C, Hudry J, Zahar S, Makwana A, Schneider N (2021). Soothing effect of an edible teether: a pilot study in children during primary dentition age. Int J Clin Pediatr Dent.

[26] Gebreyesus A, Tesfay K (2024). Effect of maternal education on completing childhood vaccination in Ethiopia: systematic review and meta-analysis. Sci Rep.

[27] ChatGPT.

[28] Google Notebook.

