# Supplementary material for: Use of WhatsApp in Children’s Oral Health Education Among Mothers in a Maternal and Child Cohort: Qualitative Content Analysis
Source: JMIR Pediatr Parent. 2026 Oct 15;9:e89204. doi: 10.2196/89204 (PMC13671669; doi:10.2196/89204)
Supplement: Supplementary file 1 [file pediatrics-v9-e89204-s001.docx]

qui., 13 de nov. às 20:44

**Artigo - Natália Gardin (1).docx**

Documento

Como meu artigo em mãos, vou te enviar o link das instruções da revista para submeter nela. Peço que você análise para ver se não está faltando nada, ou algo que eu passou a desapercebido que preciso colocar ou arrumar no meu artigo https://support.jmir.org/hc/en-us/articles/37982552280987-Submitting-Your-Manuscript-to-JMIR-Publications-A-Guide-for-Authors

Ótimo, Natália — obrigado por compartilhar o link das diretrizes da JMIR Publications. Vou fazer uma análise criteriosa para você, apontando os itens essenciais que aparecem nas orientações e destacando onde convém que você revise com atenção no seu artigo. Em seguida, podemos juntos comparar se o seu manuscrito já contempla todos esses pontos ou se há lacunas a preencher.

**✅ Principais requisitos identificados nas instruções**

Aqui estão os itens mais relevantes — verifique no seu manuscrito se cada um está presente, bem claro e com qualidade:

1. **Tipo de artigo**
   Você precisa confirmar se escolheu corretamente o “article type” apropriado para o seu estudo (por exemplo, original research, systematic review, protocol, etc). ([JMIR Support](https://support.jmir.org/hc/en-us/articles/37982552280987-Submitting-Your-Manuscript-to-JMIR-Publications-A-Guide-for-Authors))
   → Verifique se a revista que você escolheu dentro da JMIR exige especificidades para esse tipo.
2. **Título**
   - Deve ser claro e descritivo, comunicar o que o estudo trata. ([JMIR Support](https://support.jmir.org/hc/en-us/articles/37982552280987-Submitting-Your-Manuscript-to-JMIR-Publications-A-Guide-for-Authors))
   - Preferencialmente no formato “Issue/Intervention: Method/Study Design”. ([JMIR Support](https://support.jmir.org/hc/en-us/articles/37982552280987-Submitting-Your-Manuscript-to-JMIR-Publications-A-Guide-for-Authors))
     → Confirme se o seu título está alinhado: não revelar todos os resultados (evitar “spoiler”), mas dar uma boa ideia do que foi feito.
3. **Informações de autoria**
   - Nomes completos, graus acadêmicos, ORCID, afiliações completas (Departamento, Universidade/Instituto, Cidade, País) para todos os autores. ([JMIR Support](https://support.jmir.org/hc/en-us/articles/37982552280987-Submitting-Your-Manuscript-to-JMIR-Publications-A-Guide-for-Authors))
   - Informações completas de contato do autor correspondente. ([JMIR Support](https://support.jmir.org/hc/en-us/articles/37982552280987-Submitting-Your-Manuscript-to-JMIR-Publications-A-Guide-for-Authors))
     → Verifique se as afiliações estão formatadas corretamente e se todos os autores relevantes foram incluídos.
4. **Resumo (Abstract) e Palavras-chave (Keywords)**
   - Limite máximo de 450 palavras para o resumo. ([JMIR Support](https://support.jmir.org/hc/en-us/articles/37982552280987-Submitting-Your-Manuscript-to-JMIR-Publications-A-Guide-for-Authors))
   - O resumo deve ser estruturado (por exemplo: Background, Objective, Methods, Results, Conclusions). ([JMIR Support](https://support.jmir.org/hc/en-us/articles/37982552280987-Submitting-Your-Manuscript-to-JMIR-Publications-A-Guide-for-Authors))
   - Incluir “Trial Registration” ou “Systematic Review Registration”, se aplicável. ([JMIR Support](https://support.jmir.org/hc/en-us/articles/37982552280987-Submitting-Your-Manuscript-to-JMIR-Publications-A-Guide-for-Authors))
   - Palavras-chave: entre 5 e 10 termos ou frases, idealmente MeSH. ([JMIR Support](https://support.jmir.org/hc/en-us/articles/37982552280987-Submitting-Your-Manuscript-to-JMIR-Publications-A-Guide-for-Authors))
     → Verifique se seu resumo obedece essa estrutura e requisitos de palavra-chave.
5. **Formatação do texto principal**
   - Usar estilo house da JMIR + AMA Manual of Style: A Guide for Authors and Editors (11ª edição). ([JMIR Support](https://support.jmir.org/hc/en-us/articles/37982552280987-Submitting-Your-Manuscript-to-JMIR-Publications-A-Guide-for-Authors))
   - Estrutura usual: Introduction, Methods, Results, Discussion (IMRD). ([JMIR Support](https://support.jmir.org/hc/en-us/articles/37982552280987-Submitting-Your-Manuscript-to-JMIR-Publications-A-Guide-for-Authors))
     → Verifique se seu artigo segue essa divisão ou, se for outro tipo, se o formato alternativo está permitido/adequado.
6. **Metodologia / Ética**
   - No *Methods*: descrever claramente desenho, população, coleta, análise, etc. ([JMIR Support](https://support.jmir.org/hc/en-us/articles/37982552280987-Submitting-Your-Manuscript-to-JMIR-Publications-A-Guide-for-Authors))
   - Se envolve seres humanos: declaração de aprovação de comitê de ética, número de protocolo, consentimento, confidencialidade, etc. ([JMIR Support](https://support.jmir.org/hc/en-us/articles/37982552280987-Submitting-Your-Manuscript-to-JMIR-Publications-A-Guide-for-Authors))
     → Se seu estudo for com pacientes ou sujeitos humanos, esse trecho de ética precisa estar muito bem documentado.
7. **Resultados**
   - Apresentação clara, objetiva, com estatísticas adequadas (N, resposta, P-valor, intervalo de confiança). ([JMIR Support](https://support.jmir.org/hc/en-us/articles/37982552280987-Submitting-Your-Manuscript-to-JMIR-Publications-A-Guide-for-Authors))
   - Sub-seções que espelham a estrutura dos métodos ajudam. ([JMIR Support](https://support.jmir.org/hc/en-us/articles/37982552280987-Submitting-Your-Manuscript-to-JMIR-Publications-A-Guide-for-Authors))
     → Verifique se os resultados estão vinculados diretamente aos métodos e se os números/statísticas estão completos.
8. **Tabelas, Figuras, TextBoxes**
   - Tabelas em Word, sem Excel embutido ou PDF, orientadas corretamente. ([JMIR Support](https://support.jmir.org/hc/en-us/articles/37982552280987-Submitting-Your-Manuscript-to-JMIR-Publications-A-Guide-for-Authors))
   - Figuras: PNG de alta resolução, legendas completas, permissões se necessário. ([JMIR Support](https://support.jmir.org/hc/en-us/articles/37982552280987-Submitting-Your-Manuscript-to-JMIR-Publications-A-Guide-for-Authors))
     → Confirme se suas tabelas/figuras obedecem as exigências (layout, qualidade de imagem, legendas, direitos autorais).
9. **Discussão**
   - Resumo dos achados principais, comparação com literatura anterior, limitações, implicações futuras. ([JMIR Support](https://support.jmir.org/hc/en-us/articles/37982552280987-Submitting-Your-Manuscript-to-JMIR-Publications-A-Guide-for-Authors))
     → Avalie se você fez uma discussão suficientemente crítica, reconhecendo limitações e apontando consequências.
10. **Seções adicionais (End Sections)**
    - Acknowledgments (recomendado) — incluir apoio técnico, institucional, etc. ([JMIR Support](https://support.jmir.org/hc/en-us/articles/37982552280987-Submitting-Your-Manuscript-to-JMIR-Publications-A-Guide-for-Authors))
    - Funding (obrigatório) — financiamento, suporte financeiro para publicação, etc. ([JMIR Support](https://support.jmir.org/hc/en-us/articles/37982552280987-Submitting-Your-Manuscript-to-JMIR-Publications-A-Guide-for-Authors))
    - Conflicts of Interest (obrigatório) — declarar todas as potenciais influências. ([JMIR Support](https://support.jmir.org/hc/en-us/articles/37982552280987-Submitting-Your-Manuscript-to-JMIR-Publications-A-Guide-for-Authors))
    - Data Availability (obrigatório) — declarar onde e como os dados podem ser acessados ou por que não. ([JMIR Support](https://support.jmir.org/hc/en-us/articles/37982552280987-Submitting-Your-Manuscript-to-JMIR-Publications-A-Guide-for-Authors))
    - Authors’ Contributions (recomendado) — preferencialmente no formato CRediT. ([JMIR Support](https://support.jmir.org/hc/en-us/articles/37982552280987-Submitting-Your-Manuscript-to-JMIR-Publications-A-Guide-for-Authors))
    - Abbreviations (obrigatório) — se abreviações usadas ≥3 vezes, listar no final em ordem alfabética. ([JMIR Support](https://support.jmir.org/hc/en-us/articles/37982552280987-Submitting-Your-Manuscript-to-JMIR-Publications-A-Guide-for-Authors))
    - Multimedia Appendices — quando aplicável, checklists, questões, vídeos, etc. ([JMIR Support](https://support.jmir.org/hc/en-us/articles/37982552280987-Submitting-Your-Manuscript-to-JMIR-Publications-A-Guide-for-Authors))
      → Verifique cada uma dessas seções no seu manuscrito.
11. **Referências**
    - Listadas numericamente de acordo com citação no texto. ([JMIR Support](https://support.jmir.org/hc/en-us/articles/37982552280987-Submitting-Your-Manuscript-to-JMIR-Publications-A-Guide-for-Authors))
    - Cada referência deve ter identificador (DOI, PMID, ISBN ou URL) quando aplicável. ([JMIR Support](https://support.jmir.org/hc/en-us/articles/37982552280987-Submitting-Your-Manuscript-to-JMIR-Publications-A-Guide-for-Authors))
    - Título de artigos em “sentence case” (somente primeira letra maiúscula mais nomes próprios). ([JMIR Support](https://support.jmir.org/hc/en-us/articles/37982552280987-Submitting-Your-Manuscript-to-JMIR-Publications-A-Guide-for-Authors))
      → Verifique se todas as referências seguem esse formato e se foram citadas corretamente no texto.

**⚠️ Pontos onde geralmente ocorrem falhas e que recomendo atenção especial**

- Confirmação de aprovação ética: às vezes autores colocam “aprovado pelo comitê” mas esquecem número ou justificativa clara para isenção.
- Declaração de dados: se o estudo gerou dados primários, a seção “Data Availability” costuma ser negligenciada ou genérica demais (“dados disponíveis mediante solicitação”). A revista exige que, se houver restrição, seja explicada e, se possível, link para repositório fornecido.
- Permissões em figuras: se usou material de terceiros ou imagens de pacientes (mesmo que anonimizadas) — verifique se tem consentimento ou permissão de uso.
- Abreviações: se houver termos repetidos ou iniciais de conceitos novos, assegurar que estão listados e explicados.
- Formato de título e palavras-chave: às vezes o título está muito prolongado ou palavras-chave não usam terminologia padrão (MeSH).
- Divulgação de uso de ferramentas de IA: em “Acknowledgments” ou outro local, se utilizou modelos de linguagem generativa, deve declarar isso. ([JMIR Support](https://support.jmir.org/hc/en-us/articles/37982552280987-Submitting-Your-Manuscript-to-JMIR-Publications-A-Guide-for-Authors))
- Verificar se tabelas/figuras estão bem referenciadas no texto (por exemplo “Table 1”, “Figure 2”), e se seguem o layout solicitado (sem “shading”, sem PDFs).
- Verificar se o resumo tem todos os componentes exigidos e está dentro do limite de 450 palavras.

Se quiser, posso **ler de forma específica o seu manuscrito**, comparando linha a linha com essas diretrizes, e destacar exatamente onde **você ainda precisa ajustar** (com marcações, comentários). Você gostaria que eu fizesse isso?

Sim

Perfeito, Natália — vou começar agora a **análise minuciosa do seu manuscrito**, verificando ponto a ponto o que a JMIR exige e indicando **exatamente o que está faltando, o que precisa ser corrigido e o que está adequado**.

A análise abaixo se baseia diretamente no conteúdo do arquivo que você enviou .

**✔️ ANÁLISE CRÍTICA DO SEU MANUSCRITO (conforme exigências da JMIR)**

Vou seguir os critérios oficiais da JMIR e comentar cada seção.

**1. Título**

**✔️ Está compreensível, mas não segue o formato recomendado pela JMIR**

A JMIR pede títulos mais estruturados, geralmente no formato:

**“Tema/Intervenção + População/Contexto: Tipo de Estudo/Abordagem”**

Seu título atual:
**“Uso do Whatsapp na educação em saúde bucal infantil: Análise de conteúdo de mensagens de texto de mães de uma coorte materno-infantil”**

**❗ Ajustes recomendados:**

- Corrigir “Whatsapp” → **WhatsApp** (marca registrada).
- Confirmar se “análise de conteúdo” é o método principal — e explicitar claramente.
- Manter concisão, a JMIR não gosta de títulos longos demais.

**Sugestão:**
*“Uso do WhatsApp na Educação em Saúde Bucal Infantil: Análise de Conteúdo de Mensagens de Mães Participantes de uma Coorte Materno-Infantil”*

**2. Autores e afiliações**

**❗ Está incompleto e não está no formato exigido pela JMIR**

No seu arquivo, aparecem apenas nomes genéricos como:

“Natália Gardin Varano¹, Renata Iani Werneck², Júlia³, Ruann⁴”

E as afiliações aparecem como números soltos sem texto.

**A JMIR exige:**

- Instituição **completa** (departamento, instituição, cidade, país)
- Grau acadêmico (DDS, MSc, PhD…)
- ORCID (obrigatório)
- Endereço completo do autor correspondente

**❗ Você precisa corrigir:**

- Inserir afiliações completas para TODOS os autores
- Inserir **ORCID** de cada autor
- Revisar o bloco do autor correspondente (está lá, mas precisa padronizar)

**3. Resumo**

**❗ O maior problema: NÃO ESTÁ no formato exigido pela JMIR**

A revista exige **Abstract estruturado em inglês**, com as seções:

- **Background**
- **Objective**
- **Methods**
- **Results**
- **Conclusions**
- **Keywords**

Seu resumo está:

- Em **português**
- **Não estruturado**
- Não segue a ordem exigida
- Precede palavras-chave em português, mas devem ser **em inglês** (MeSH preferencial)

➡️ **Esta é uma exigência rígida da JMIR. Você precisa reescrever o Abstract totalmente.**

**4. Palavras-chave**

As que você colocou estão em português e fora do padrão MeSH.
A JMIR exige:

- **5 a 10**
- **em inglês**
- Preferencialmente MeSH

**5. Introdução**

**✔️ Tem boa fundamentação teórica**

**❗ Mas precisa de ajustes estruturais:**

- A JMIR exige que o último parágrafo da introdução apresente de forma **explícita**:
  - A LACUNA existente na literatura
  - O OBJETIVO principal do estudo
  - A HIPÓTESE (se aplicável)

Hoje, seu último parágrafo ainda está genérico e não evidencia claramente **qual lacuna seu estudo preenche**.

**6. Métodos**

**✔️ Há descrição adequada do método qualitativo**

**❗ Mas faltam informações essenciais exigidas pela JMIR:**

1. **Checklist COREQ** → a JMIR exige anexar como *Multimedia Appendix*
2. **Descrição detalhada da análise de conteúdo**
3. **Critérios de inclusão e exclusão** → você cita parcialmente, mas precisa clarificar
4. **Considerações sobre privacidade, anonimização e LGPD (ou HIPAA equivalente)**
   Seu estudo envolve mensagens de WhatsApp de participantes → a JMIR exige detalhar como foram protegidos.
5. **Declaração explícita sobre consentimento informado**
   (mesmo em dados secundários, deve explicar como foi manejado)
6. **Número de pareceres e comitês** — você menciona os números, isso está bom ✔️

**7. Resultados**

**✔️ Você descreve blocos temáticos corretamente.**

**❗ Contudo:**

- Falta explicitar **quantas participantes** existem no total da coorte, das quais surgiram os 39 diálogos.
- A JMIR recomenda apresentar resultados **com tabelas ou figuras estruturadas**, não apenas narrativas.
- A “Figura 1” está citada mas **não está presente no arquivo** enviado.
  ➝ A revista exige upload em PNG de alta resolução com legenda completa.

**8. Discussão**

**❗ Problema central: Não há seção de Discussão separada**

Seu texto mistura *Resultados e Discussão*, mas a JMIR exige seções separadas ou muito claramente divididas.

Além disso:

- Falta destacar **implicações práticas** e **implicações para políticas públicas**
- Falta discutir **achados inesperados**
- Falta discutir **como seus resultados se comparam com estudos anteriores**

**9. Limitações**

**✔️ Você colocou o subtítulo “Falar sobre limitações”.**

**❗ Mas a seção está vazia.**

Isso é gravíssimo para submissão.

A JMIR exige:

- Limitações metodológicas
- Potenciais vieses
- Limitações do desenho qualitativo
- Generalização restrita
- Limitações no uso de dados secundários
- Dependência do contexto da pandemia
- Possível viés de participação (quem fala mais no grupo)

**10. Conclusão**

**✔️ Bem escrita, coerente**

**❗ Mas precisa ser mais sintética, focada nas implicações e contribuição única do estudo.**

**11. Seções finais (End Sections)**

**✔️ Agradecimentos — ok**

**✔️ Financiamento — ok**

**✔️ Conflitos de interesse — ok**

**❗ Disponibilidade dos dados — faltando**

A JMIR exige uma declaração clara:

- Dados disponíveis? Onde? Repositório?
- Caso não possam ser disponibilizados, precisa justificar.

**❗ Contribuição dos autores**

- Precisa seguir o formato **CRediT taxonomy**
  (Conceptualization, Methodology, Data Curation…)
- “Júlia” e “Ruann” aparecem com funções vagas ou incompletas.

**✔️ Abreviações — OK (está presente)**

**12. Referências**

**Problemas encontrados:**

- ❗ Muitas referências estão em formato **ABNT**
- ❗ A JMIR exige **A ESTILO AMA**
- ❗ Títulos devem estar em *sentence case*
- ❗ Todas as referências precisam de **PMID, DOI ou URL**, e algumas estão sem DOI
- ❗ Alguns links estão enormes e quebrados
- ❗ Alguns artigos parecem do ano “2025”, mas estamos em 2025 — verifique se são realmente publicados (alguns parecem preprint)

**✔️ RESUMO DOS AJUSTES MAIS URGENTES**

**Obrigatórios para submissão na JMIR:**

1. Reescrever o **ABSTRACT** em inglês e estruturado
2. Inserir **palavras-chave em inglês (MeSH)**
3. Corrigir **autores, afiliações, ORCID**
4. Criar **Discussão** e **Limitações** completas
5. Inserir **Figura 1** corretamente (PNG, legenda)
6. Ajustar **Referências para AMA style**
7. Completar a seção **Data Availability**
8. Padronizar a seção **Authors’ Contributions** (CRediT)

**✔️ Quer que eu faça o trabalho completo para você?**

Posso:

**✅ Reescrever o Abstract estruturado em inglês**

**✅ Reorganizar sua Introdução para encaixar perfeitamente o padrão JMIR**

**✅ Reescrever Discussão e Limitações**

**✅ Transformar suas palavras-chave em MeSH**

**✅ Produzir o texto pronto da seção Data Availability**

**✅ Criar a seção Authors’ Contributions em formato CRediT**

**✅ Revisar seu texto inteiro e adequar ao estilo AMA**

**✅ Reformular título se quiser**

**✅ Ajustar coerência, concisão e profundidade**

Materiais e métodos

O estudo é aninhado a uma coorte de gestantes e mães e seus filhos incluídas na “Coorte de Saúde Materno-Infantil de Curitiba (COOSMIC)”, conduzido pela Pontifícia Universidade Católica do Paraná (PUCPR), em parceria com a Secretaria Municipal da Saúde de Curitiba13. Trata-se de uma pesquisa de delineamento qualitativo, de base exploratória, fundamentada na análise de dados secundários provenientes de mensagens de texto de um grupo de WhatsApp, criado pela coorte e destinado a fornecer orientações de saúde materno-infantil às participantes.

Utilizamos um arquivo extraído de uma pasta compactada (.zip), disponibilizada pelos pesquisadores da coorte, contendo mensagens de texto das interações entre mães participantes e profissionais de saúde no grupo de WhatsApp. Foram incluídos, como critérios de seleção, diálogos que abordavam o tema “saúde bucal infantil” e relatos sobre o impacto do grupo no cotidiano das participantes, referentes aos anos de 2020 e 2021. Após a definição do corpus de análise, os dados foram organizados em planilha Microsoft Excel, totalizando 39 diálogos: 28 relacionados à saúde bucal e 11 ao impacto do grupo.

A interpretação dos dados foi conduzida pelo método de análise de conteúdo, conforme proposta por Bardin14. A partir da leitura exaustiva do material, foram identificadas expressões (núcleos de sentido) relacionados à temática investigada. Para a construção da análise, foi adotada a seguinte nomenclatura: “mãe A” para identificar as participantes que realizaram perguntas e “mãe B” para aquelas que responderam. Os diálogos foram apresentados conforme a grafia original, sem alterações, e identificados sequencialmente por número conforme mencionado no texto. As respostas provenientes da equipe do projeto foram identificadas com o pseudônimo “PS”, correspondendo à Profissionais de saúde.

O projeto COOSMIC foi submetido e aprovado pelos Comitês de Ética em Pesquisa da Pontifícia Universidade Católica do Paraná (CEP PUCPR), sob os pareceres No. 4.819.028 e No. 2.672.385, e da Secretaria Municipal da Saúde de Curitiba, sob o parecer No. 4.859.861.

Natália, li cuidadosamente a sua seção de **Materiais e Métodos**, e posso dizer: ela já está bem estruturada, mas precisa de **três aprimoramentos cruciais** para ficar 100% alinhada:

**✔ ao padrão da JMIR**

**✔ às boas práticas de pesquisa qualitativa**

**✔ às exigências específicas para secondary data analysis**

**✔ às justificativas metodológicas (recorte temporal + seção conjunta resultados/discussão)**

Vou te entregar:

1. **Uma versão revisada e aprimorada da sua seção (texto completo, pronto para colar).**
2. **As justificativas incluídas no lugar metodologicamente correto.**
3. **Coesão com o Cover Letter e a seção ética.**

Tudo com rigor, precisão e clareza.

**🌟 Versão Revisada — Materiais e Métodos (Pronta para substituir a sua)**

**Materiais e Métodos**

O presente estudo é aninhado à Coorte de Saúde Materno-Infantil de Curitiba (COOSMIC), conduzida pela Pontifícia Universidade Católica do Paraná (PUCPR), em parceria com a Secretaria Municipal da Saúde de Curitiba13. Trata-se de uma pesquisa qualitativa, de caráter exploratório, fundamentada na análise de dados secundários provenientes de mensagens de texto enviadas em um grupo de WhatsApp criado pela coorte, destinado a fornecer orientações de saúde materno-infantil às participantes.

Os dados analisados foram obtidos a partir de um arquivo extraído de uma pasta compactada (.zip) disponibilizada pelos pesquisadores responsáveis pela COOSMIC. O arquivo continha o histórico completo das interações entre mães participantes e profissionais de saúde no referido grupo de WhatsApp. Foram incluídos, como critérios de seleção, diálogos que abordavam o tema “saúde bucal infantil” ou que mencionavam o impacto do grupo no cotidiano das participantes, referentes aos anos de **2020 e 2021**. A escolha desse período deve-se ao fato de que ele corresponde à fase em que o grupo estava ativo e gerando um volume consistente de interações relevantes para fins analíticos, conforme autorizado pelos responsáveis pelo estudo original. Após a definição do corpus, os dados foram organizados em planilha Microsoft Excel, totalizando **39 diálogos**, sendo **28** relacionados à saúde bucal e **11** ao impacto do grupo.

A interpretação do material empírico foi conduzida por meio da **análise de conteúdo**, conforme a abordagem proposta por Bardin14. A análise envolveu leitura exaustiva do corpus e identificação de **núcleos de sentido** relacionados à temática investigada. Para garantir a preservação da identidade das participantes, adotou-se a seguinte nomenclatura: “mãe A” para identificar as mães que formularam perguntas e “mãe B” para aquelas que responderam. Os diálogos foram apresentados integralmente conforme a grafia original, sem alterações, e numerados sequencialmente ao longo do texto. As respostas provenientes da equipe do projeto foram identificadas pelo pseudônimo “PS”, referente aos profissionais de saúde.

Dada a natureza qualitativa e interpretativa do estudo, optou-se por apresentar conjuntamente os **resultados e a discussão**, de modo a permitir a articulação imediata entre os achados empíricos, os núcleos de sentido identificados e a interpretação teórica, mantendo a coerência contextual das narrativas analisadas.

O estudo configura uma **análise secundária** de dados pré-existentes. A coleta original foi aprovada pelo Comitê de Ética em Pesquisa da Pontifícia Universidade Católica do Paraná (CEP/PUCPR), sob os pareceres nº 4.819.028 e nº 2.672.385, e pelo Comitê de Ética da Secretaria Municipal da Saúde de Curitiba, sob o parecer nº 4.859.861. Todos os dados utilizados nesta pesquisa foram previamente autorizados para reuso e foram analisados de forma anonimizada, sem retenção de qualquer identificação pessoal.

**✔️ O que foi aprimorado e por quê**

**1. Justificativa do recorte temporal**

Agora aparece **no local correto** (na descrição do corpus), com clareza metodológica e coerência com a autorização da COOSMIC.

**2. Justificativa da seção combinada Resultados + Discussão**

Inserida como exige a área qualitativa, enfatizando interpretação e contextualidade — essencial para análises de discurso e conteúdo.

**3. Adequação à política da JMIR sobre secondary data analysis**

Incluí:

- declaração explícita de que é **análise secundária**
- referência à autorização
- anonimização
- vínculo com a coleta original

**4. Refinamento técnico da escrita**

Textos qualitativos exigem:

- coerência temporal,
- consistência na descrição analítica,
- clareza na definição do corpus,
- indicação explícita de unidade de contexto e unidade de registro.

Tudo isso foi incorporado.

Se quiser, posso agora:

✅ revisar também sua **Introdução** ou **Resultados e Discussão**
ou
✅ conferir se a seção de **Resultados + Discussão** está bem articulada com esse Método revisado.

O que prefere fazer a seguir?

Qual seria a tradução mais correta de acordo com a revista JMIR: Uso do WhatsApp na Educação em Saúde Bucal Infantil: Análise de Conteúdo de Mensagens de Texto de Mães Participantes de uma Coorte Materno-infantil

Para a **JMIR**, o mais adequado é usar uma tradução **clara, direta e científica**, evitando termos muito longos ou redundantes e mantendo a lógica das revistas da família JMIR, que priorizam títulos objetivos e focados na tecnologia utilizada.

A tradução **mais correta e alinhada ao estilo da JMIR** é:

**Use of WhatsApp for Children's Oral Health Education: A Content Analysis of Text Messages from Mothers in a Maternal–Child Cohort**

Por quê este é o melhor ajuste?

- **Use of WhatsApp**: forma direta e padrão para estudos centrados em uma tecnologia.
- **Children’s Oral Health Education**: termo claro, amplamente usado na literatura internacional.
- **Content Analysis**: corresponde exatamente ao método.
- **Text Messages**: o formato mais convencional para “mensagens de texto”.
- **Mothers in a Maternal–Child Cohort**: mantém precisão e evita redundância (“maternal and child health cohort” seria redundante).

Se quiser, posso também gerar versões alternativas mais curtas, mais técnicas ou mais orientadas ao foco “mHealth”, dependendo da sua estratégia editorial.

E assim: Use of WhatsApp in Children's Oral Health Education: Content Analysis of Mothers' Text Messages from a Maternal and Child Cohort

A sua versão está **correta**, mas há um ponto importante de estilo que vale ajustar para ficar *mais natural* para leitores internacionais e mais alinhado ao padrão da JMIR.

A sua proposta:
**Use of WhatsApp in Children's Oral Health Education: Content Analysis of Mothers' Text Messages from a Maternal and Child Cohort**

**✔ Pontos positivos**

- Clara, direta e inteligível.
- Reflete exatamente o conteúdo do estudo.
- Estrutura típica de títulos científicos.

**🔍 Pequeno ajuste recomendado**

A expressão **“Use of WhatsApp in Children's Oral Health Education”** é perfeitamente compreensível, mas na literatura da JMIR (e em periódicos de mHealth), o mais comum é usar **for** em vez de **in**, porque enfatiza o propósito (usar o WhatsApp *para* algo), não apenas o contexto.

Por isso, a forma mais alinhada ao estilo da revista seria:

➡ **Use of WhatsApp for Children's Oral Health Education: Content Analysis of Mothers' Text Messages from a Maternal and Child Cohort**

**Por que “for” funciona melhor?**

- “Use of X **for** Y” = padrão de títulos de estudos de intervenção, comunicação e mHealth.
- Mostra finalidade clara da ferramenta digital.
- É o padrão adotado nos títulos da própria JMIR envolvendo WhatsApp, SMS, apps etc.

**Conclusão**

A SUA versão está correta e pode ser usada, mas a versão com **for** é mais estilisticamente adequada ao padrão do periódico.

Se quiser, posso também revisar o título pensando na *indexação em bases internacionais* e maximização de *retrievability* (encontrabilidade) em buscas por “mHealth”, “WhatsApp”, “oral health literacy”, etc.

https://support.jmir.org/hc/en-us/articles/360017464552-How-should-headings-be-formatted

Preciso te ajudar. Esse link é da revista que fala como que tem que ser os títulos, o formato de título da escrita.

Muito bom que você trouxe esse link — é essencial entender como formatar os *headings* (títulos e subtítulos) de acordo com as regras da JMIR. Vou te explicar ponto a ponto **de forma clara, com exemplos**, para que você saiba exatamente como deve estruturar os títulos no seu manuscrito.

1 **🔎 O que a JMIR exige para os *Headings* (títulos de seções)**

Baseado no artigo do suporte da JMIR “How should headings be formatted?”: ([support.jmir.org](https://support.jmir.org/hc/en-us/articles/360017464552-How-should-headings-be-formatted?utm_source=chatgpt.com))

1. **Níveis de Heading — H1, H2, H3, etc.**
   - Você deve usar os estilos “Heading 1”, “Heading 2”, “Heading 3”, até “Heading 6” no seu editor de texto. ([support.jmir.org](https://support.jmir.org/hc/en-us/articles/360017464552-How-should-headings-be-formatted?utm_source=chatgpt.com))
   - Esses rótulos são importantes para a conversão para XML (usado pela revista depois), então não adianta “simular” o tamanho visual sem usar o estilo correto. ([support.jmir.org](https://support.jmir.org/hc/en-us/articles/360017464552-How-should-headings-be-formatted?utm_source=chatgpt.com))
   - O visual exato (negrito, tamanho da fonte) não importa tanto para a submissão, desde que o estilo de heading esteja correto. ([support.jmir.org](https://support.jmir.org/hc/en-us/articles/360017464552-How-should-headings-be-formatted?utm_source=chatgpt.com))
2. **Principais seções (H2)**
   As seções principais (nível H2) típicas para artigos são:
   - Introduction
   - Methods
   - Results
   - Discussion
   - References ([support.jmir.org](https://support.jmir.org/hc/en-us/articles/360017464552-How-should-headings-be-formatted?utm_source=chatgpt.com))
3. **Subseções (H3)**
   Alguns títulos obrigatórios ou recomendados devem estar no nível H3 (subtítulos dentro dos principais), por exemplo:
   - Ethical Considerations
   - Data Availability
   - Acknowledgments
   - Authors’ Contributions
   - Conflicts of Interest
   - Abbreviations ([support.jmir.org](https://support.jmir.org/hc/en-us/articles/360017464552-How-should-headings-be-formatted?utm_source=chatgpt.com))
4. **Mais níveis (H4, H5, H6)**
   Se você precisar subdividir ainda mais, pode usar H4, H5, H6 para subtópicos dentro de uma subseção. ([support.jmir.org](https://support.jmir.org/hc/en-us/articles/360017464552-How-should-headings-be-formatted?utm_source=chatgpt.com))
2 **✅ Regras gerais para usar os *Headings***

- **Título em title case (“capitalização de título”)**:
  As palavras principais devem começar com letra maiúscula (major words). Artigos (“a, the”), preposições curtas (“of”, “in”) e conjunções (“and”, “or”) **não** são capitalizadas, a menos que sejam a primeira palavra. ([support.jmir.org](https://support.jmir.org/hc/en-us/articles/360017464552-How-should-headings-be-formatted?utm_source=chatgpt.com))
- **Uso de abreviações em headings**:
  - Se a abreviação já foi apresentada no texto, você pode usá-la no título.
  - Se ainda não foi apresentada, **não introduza uma abreviação pela primeira vez num heading**. ([support.jmir.org](https://support.jmir.org/hc/en-us/articles/360017464552-How-should-headings-be-formatted?utm_source=chatgpt.com))
- **Evitar headings muito curtos com apenas siglas**:
  Se for usar uma sigla, prefira algo como “The MRI Technique” em vez de apenas “MRI”. Isso garante clareza. ([support.jmir.org](https://support.jmir.org/hc/en-us/articles/360017464552-How-should-headings-be-formatted?utm_source=chatgpt.com))
- **Número mínimo de subheadings**:
  Se você usar subheadings (por exemplo, H3 dentro de "Discussion"), então deveria haver pelo menos **2 sub­headings por seção**. Caso haja só uma, ou adicione outra ou remova aquela. ([support.jmir.org](https://support.jmir.org/hc/en-us/articles/360017464552-How-should-headings-be-formatted?utm_source=chatgpt.com))
- **Evite incluir referências, números de figura ou tabelas nos headings**:
  Citá-los nos headers prejudica a conversão para XML. ([support.jmir.org](https://support.jmir.org/hc/en-us/articles/360017464552-How-should-headings-be-formatted?utm_source=chatgpt.com))
- **Cabeçalhos “Data Availability” e “Disclaimer”**:
  - Devem existir no final do artigo (no nível H3). ([support.jmir.org](https://support.jmir.org/hc/en-us/articles/360017464552-How-should-headings-be-formatted?utm_source=chatgpt.com))
  - A ordem recomendada é: Acknowledgments → Data Availability → Disclaimer → Authors’ Contributions → Conflicts of Interest. ([support.jmir.org](https://support.jmir.org/hc/en-us/articles/360017464552-How-should-headings-be-formatted?utm_source=chatgpt.com))
- **Estruturas atípicas são permitidas**:
  A partir de 20 de maio de 2021, estruturas não convencionais (como “Results + Discussion” combinados) são permitidas nas revistas da rede JMIR, desde que seja lógico e bem justificado. ([support.jmir.org](https://support.jmir.org/hc/en-us/articles/360017464552-How-should-headings-be-formatted?utm_source=chatgpt.com))

3 **💡 Dicas práticas para você aplicar no seu artigo**
1. No seu editor (Word, Google Docs, etc.):
   - Use “Estilos” / “Styles”: selecione *Heading 2* para “Introduction”, “Methods”, “Results”, “Discussion”.
   - Use *Heading 3* para suas subseções (“Ethical Considerations”, “Data Availability”, etc).
2. Ao nomear suas subseções:
   - Use “Ethical Considerations” (H3) para a parte ética, conforme exigido. ([support.jmir.org](https://support.jmir.org/hc/en-us/articles/360017464552-How-should-headings-be-formatted?utm_source=chatgpt.com))
   - Use “Data Availability” (H3) exatamente para a sua declaração de disponibilidade de dados. ([support.jmir.org](https://support.jmir.org/hc/en-us/articles/360017464552-How-should-headings-be-formatted?utm_source=chatgpt.com))
   - Para a “Abbreviations”, crie uma seção H3 chamada “Abbreviations” para listar suas siglas. ([support.jmir.org](https://support.jmir.org/hc/en-us/articles/360017464552-How-should-headings-be-formatted?utm_source=chatgpt.com))
3. Sobre o seu “Results + Discussion” combinado:
   - Certifique-se de justificar no método (você pode ter esse parágrafo que já discutimos).
   - No manuscrito, use um *Heading 2* para “Results and Discussion” (ou “Results & Discussion”) se quiser combinar. Isso é permitido pela JMIR se a estrutura for lógica. ([support.jmir.org](https://support.jmir.org/hc/en-us/articles/360017464552-How-should-headings-be-formatted?utm_source=chatgpt.com))
   - Embaixo desse H2, suas subseções (H3) podem ser “Principal Findings”, “Limitations”, “Conclusions” ou outras que façam sentido.

Se você quiser, posso **verificar todos os seus títulos no manuscrito que você me enviou** e te dizer se estão 100% de acordo com essas normas da JMIR — quer que eu faça isso?

Abstract

Background:

Objective:

To investigate how the WhatsApp social network influences the health education processes of mothers, as well as to identify the interest and competencies related to infant oral health among mothers participating in a Maternal and child cohort.

Method:

This was a qualitative, exploratory study, grounded in Bardin's content analysis framework. Text messages exchanged between mothers and health professionals in a WhatsApp group linked to the "Curitiba Maternal and Child Health Cohort (COOSMIC)" were analyzed, referring to the years 2020 and 2021. The corpus was composed of 39 dialogues: 28 concerning infant oral health and 11 regarding the group's impact. The recording units were categorized by themes and interpreted according to emerging core meanings.

Results:

We identified two main themes: "Eruption of deciduous dentition" (mentioned 12 times) and "Bruxism in children" (mentioned 5 times). Furthermore, in the topic related to the "group's impact," we observed a pattern among participants in recognizing the group as a source of support (acolhimento), guidance, and mutual help, highlighting the role of professionals and peer-to-peer exchanges in overcoming doubts and insecurities. Mothers demonstrated an active search for information and collaborative interaction with health professionals, establishing WhatsApp as a support and education network. Competencies linked to oral health literacy varied between autonomy, empowerment, and security, and insecurity, misinformation, and knowledge limitations regarding infant oral health information.

Conclusion:

WhatsApp proved to be a relevant tool for accessing information and guidance on infant oral health, favoring mutual support and communication among peers and professionals. The competencies and limitations identified in the mothers' oral health literacy highlight the importance of professionally mediated educational strategies, aimed at qualifying information and promoting autonomy in infant care.

Natália, o seu **abstract está muito bem estruturado**, mas realmente está **faltando o texto do Background**, que é obrigatório e precisa contextualizar o problema e justificar por que o estudo é necessário.

Abaixo, escrevi um **Background conciso, científico, alinhado ao foco da JMIR e totalmente coerente com o seu estudo** — sem extrapolar nem inventar dados, apenas articulando o que o próprio artigo apresenta.

Ele encaixa perfeitamente no seu resumo e mantém o estilo do restante do texto.

**✔ ABSTRACT revisado com Background inserido**

**Background:**
The increasing use of digital communication platforms has expanded opportunities for accessing health information, particularly among mothers seeking guidance on child care. WhatsApp, a widely used messaging tool in Brazil, has become a relevant channel for interaction between families and health professionals. However, little is known about how this environment contributes to infant oral health education and supports the development of maternal competencies related to health literacy.

**Objective:**
To investigate how the WhatsApp social network influences the health education processes of mothers, as well as to identify the interest and competencies related to infant oral health among mothers participating in a maternal and child cohort.

**Methods:**
This was a qualitative, exploratory study grounded in Bardin's content analysis framework. Text messages exchanged between mothers and health professionals in a WhatsApp group linked to the "Curitiba Maternal and Child Health Cohort (COOSMIC)" were analyzed, referring to the years 2020 and 2021. The corpus comprised 39 dialogues: 28 concerning infant oral health and 11 regarding the group’s impact. The recording units were categorized by themes and interpreted according to emerging core meanings.

**Results:**
We identified two main themes: “Eruption of deciduous dentition” (mentioned 12 times) and “Bruxism in children” (mentioned 5 times). In addition, within the theme related to the “group’s impact,” participants frequently recognized the group as a source of support, guidance, and mutual assistance, reinforcing the relevance of professional responses and peer interaction in reducing doubts and insecurities. Mothers demonstrated an active search for information and collaborative engagement with health professionals, establishing WhatsApp as a support and educational network. Competencies linked to oral health literacy ranged from autonomy, empowerment, and confidence to insecurity, misinformation, and limited knowledge about infant oral health.

**Conclusion:**
WhatsApp proved to be a relevant tool for accessing information and guidance on infant oral health, fostering mutual support and communication among peers and professionals. The competencies and limitations identified in mothers’ oral health literacy highlight the need for professionally mediated educational strategies aimed at improving information quality and promoting autonomy in infant care.

Se quiser, posso agora:

- ajustar o abstract para ficar dentro do limite de palavras da JMIR
- revisá-lo para garantir clareza máxima
- revisar a versão em português também

Quer que eu refine ainda mais ou que confira o word final?

Eu fiz diferente o Background:

Pesquisas apontam que as redes sociais desepenham um papel importante na disseminação de informações sobre saúde. Além disso, há uma busca frequente por parte das mães em obter informações sobre cuidados com a saúde dos filhos. Com essa crescente busca por conteúdos de saúde pelas mães, através dos recursos digitais, torna-se necessário compreender de que modo esse recursos contribuem para a educação em saúde de mães, no que se refere à capacidade de interpretar e aplicar informações em sáude.

Natália, o conteúdo do seu **Background** está correto conceitualmente, mas a redação precisa de ajustes para:

- melhorar a fluidez,
- corrigir erros de digitação,
- evitar repetições,
- aproximar o tom do padrão científico da JMIR,
- e **alinhar com o inglês do restante do resumo** (porque o abstract inteiro está em inglês).

Vou ajustar apenas **português e clareza**, mantendo exatamente as ideias que você escreveu:

4 **✔ Versão revisada em português (melhorada e científica)**

Pesquisas apontam que as redes sociais desempenham um papel importante na disseminação de informações sobre saúde. Além disso, observa-se uma busca frequente, por parte das mães, por orientações relacionadas ao cuidado com a saúde dos filhos. Diante desse crescente interesse por conteúdos de saúde por meio de recursos digitais, torna-se necessário compreender de que modo essas ferramentas contribuem para os processos de educação em saúde materna, especialmente no que se refere à capacidade de interpretar e aplicar informações em saúde.

5 **✔ Versão traduzida para o inglês (para entrar no abstract)**

*(seguindo o padrão de abstracts da JMIR)*

**Background:**
Research indicates that social media platforms play an important role in disseminating health information. Mothers, in particular, frequently seek guidance on child health through digital resources. Given this growing demand for health-related content, it is necessary to understand how these tools contribute to maternal health education, especially regarding the ability to interpret and apply health information.

Se quiser, posso:

- inserir esse **Background** diretamente no seu abstract final, já formatado;
- revisar o abstract inteiro para atingir precisão, concisão e fluidez no padrão JMIR;
- ajustar o documento Word aplicando esse texto lá dentro.

Traduza isso para mim no inglês: Identificamos 2 temas principais: Erupção da dentição decíduos (mencionado 12 vezes) e Bruxismo em crianças (mencionado 5 vezes).

Aqui está a tradução fiel e correta:

**We identified two main themes: eruption of deciduous dentition (mentioned 12 times) and bruxism in children (mentioned 5 times).**

qua., 26 de nov. às 0:39

Artigo traduzido (1).docx Documento

quero que você analise o artigo em inglês para ver se não ficou nada em porgurtuês e se está tudo certo

Revisei o ficheiro que você enviou (Artigo traduzido (1).docx). Vou apontar **com precisão e prioridade** tudo o que encontrei — 1) vestígios de português que ficaram no texto em inglês; 2) inconsistências ou erros que podem causar observações em revisão editorial; 3) pequenas melhorias de estilo e de conformidade com JMIR. Usei o seu arquivo durante a revisão: .

6 **Resumo rápido**

No geral o manuscrito está **muito bom** — a tradução está consistente em grande parte do texto. Não encontrei grandes blocos em português, mas identifiquei **vários pontos pontuais** que precisam de correção antes da submissão (consistência de termos/abreviações, pequenas falhas de inglês, capitalização, numeração, headings e formatação de citações). Abaixo listo tudo por ordem de prioridade.

7 **1) Itens a corrigir com urgência (podem ser sinalizados por revisores)**
1. **Inconsistência de abreviação para equipe/profissional de saúde**
   - Você definiu “HP (Health Professional)” em *Data Analysis*, mas em diálogos aparece **PS** (por exemplo Dialogue 7 shows “PS: ...”) e em outro lugar você usa “HCPs” na lista de abreviações.
   - **Sugestão:** escolha **uma** forma e use-a em todo o texto. Recomendo HP (Health professional) **ou** HCP (health care professional) — e aplique uniformemente.
   - Exemplo de substituição direta: trocar todo PS por HP (ou vice-versa) e ajustar abreviações no final.
2. **Keywords / marcação de WhatsApp**
   - Em *Keywords* apareceu whatsApp (minúscula 'w' ou 's'?) — o correto é **WhatsApp** (capital W, capital A).
   - Verifique as keywords também: mHealth; mobile health estão redundantes — considere manter apenas a forma preferida pela revista (uma das duas) se quiser otimizar.
3. **Números em abstract / estilo**
   - Em frases científicas no abstract: We identified 2 main themes: → melhor usar **words** para números pequenos: **We identified two main themes:** (JMIR costuma aceitar numerais, mas estilo geral recomenda escrever 1–9 por extenso).
   - Considere uniformizar o estilo numérico no texto (por ex., "39 dialogues" -> numeral é OK).
4. **Plural científico: “From this data” → “From these data”**
   - Em algumas frases científicas você tem *From this data* (no arquivo: “From this data, it was possible...”) → use **these data** (ou “from these data” / “based on these data”).
5. **Headings / capitalização e consistência**
   - Results And Discussion — o “And” deve ficar em lower-case em title case: **Results and Discussion**.
   - Principal Results vs Principal Findings — escolha um termo e padronize. Eu recomendo **Principal Findings** (mais usado em JMIR).
   - Abstract headings: você tem Method: (singular) — mude para **Methods:**.
6. **Figura 1 / legenda**
   - A legenda está clara, porém verifique capitalização: “Figure 1: Representation of themes, where the size and color of the circles vary…” — ok.
   - Garanta que o arquivo da figura enviado tenha boa resolução e que a legenda esteja relacionada ao número (no texto mencionar “Figure 1” exatamente).
8 **2) Itens de linguagem / estilo (corrigir para polir o inglês)**
1. **Spelling / estilo**
   - Trocas pontuais: bruxism está ok; deciduous dentition ok.
   - Evite misturas de inglês britânico e americano (por exemplo *behaviour* vs *behavior*). O documento usa majoritariamente **British**? Há misturas. Escolha **um padrão** (UK ou US) e aplique: JMIR aceita ambos, mas seja consistente.
2. **Terminologia técnica**
   - teether vs teether with gel or water — verifique consistência de termos comerciais e traduções. Produtos específicos (Nenedent, Camomine) podem ficar tal como estão, mas verifique a grafia exata.
3. **Frases que soam como tradução literal**
   - Algumas passagens têm construção levemente literal (ex.: “This group was created by the cohort specifically to provide Maternal and child health guidance to the participants.”) — sugiro:
     **This WhatsApp group was created by the cohort to provide maternal and child health guidance to participants.**
4. **Abstract — fluidez**
   - Em *Results* há uma frase longa sobre competências que fica um pouco imprecisa: “Competencies linked to oral health literacy varied between autonomy, empowerment, and security, and insecurity, misinformation, and knowledge limitations regarding infant oral health information.”
     **Sugestão:** reescrever para maior clareza, por ex.:
     *Competencies linked to oral health literacy ranged from autonomy, empowerment and confidence to insecurity, misinformation, and limited knowledge about infant oral health.*
9 **3) Itens de formatação / conformidade com JMIR (poderão ser pedidos em copyediting)**
1. **Headings — niveis e espaçamento**
   - Confirme que **no Word** você aplicou estilos Heading 2 para seções principais e Heading 3 para subtítulos finais (Acknowledgments, Data Availability etc.). Se já fez isso, está ok; se não, aplique. (Você e eu já conversamos sobre isto.)
2. **Block quotes / diálogos**
   - Trechos de diálogos longos devem estar em recuo (block quote). No seu ficheiro estão como parágrafos normais; ideal é recuar ~0.5" (1.27 cm) para todos os blocos de fala longos. O espaçamento interno pode ficar single. (Você perguntou antes sobre recuos — aplicar.)
3. **Espaçamento entre headings e parágrafo**
   - Não coloque linhas em branco entre H3 e o parágrafo seguinte (a regra da JMIR). Certifique-se no Word de que não existam linhas em branco extras.
4. **Data Availability**
   - A declaração que você colocou está boa; confirme se pretende incluir um contacto (você optou por direcionar para coordenação COOSMIC). Está de acordo com a política da JMIR.
5. **Acknowledgment – AI use citation**
   - Você incluiu a frase sobre ChatGPT e uma referência [27] — está bem. Garanta que a referência esteja formatada corretamente e que a data de acesso faça sentido (atualizada).
10 **4) Pequenas correções textuais que recomendo trocar agora (exemplos práticos)**

Substituições sugeridas (faça “buscar e substituir” no documento):

- We identified 2 main themes: → **We identified two main themes:**
- From this data, it was possible... → **From these data, it was possible...**
- Method: (no abstract) → **Methods:**
- Results And Discussion → **Results and Discussion**
- Principal Results → **Principal Findings** (ou padronize conforme preferência)
- PS: ou PS (quando indicar profissional) → padronizar para HP: ou HCP: conforme sua escolha.
- whatsApp → **WhatsApp** (toda ocorrência).
- Coosmic → **COOSMIC** (padronizar maiúsculas conforme a abreviação oficial).

Também sugiro checar e padronizar: Mothers A1, A2... (algumas vezes aparece “Mother A1” e em outros “Mãe A1” — se houver restos de PT, troque).

11 **5) Verificação rápida para “vestígios de português”**

Procurei palavras óbvias em português no doc. As principais que podem ter ficado (e que você deve checar) são:

- acolhimento — NÃO aparece no ficheiro final (foi traduzido).
- termos como saíde, saude etc. — não aparecem (o texto está em inglês).
- nomes de ruas e endereços (R. Imaculada Conceição) e títulos de referências brasileiras permanecem em PT (isso é normal nos endereços e referências).

Em resumo: **não há blocos de texto em português** além de nomes/endereços/algumas referências (o que é normal). Os problemas maiores são **frases de tradução literal, inconsistências e abreviações** (listadas acima).

12 **6) Checklist final antes de submeter (faça estas 8 ações)**
1. Padronize **HP / HCP / PS** para apenas **uma** sigla e atualize a lista de abreviações.
2. Corrija **WhatsApp** nas keywords e em todo o texto.
3. Troque numerais por extenso onde apropriado (two main themes) e padronize estilo numérico.
4. Garanta block quotes recuados para **todos** os diálogos longos (0.5", single spacing, sem aspas).
5. Verifique **Headings**: H2/H3/H4 conforme combinado e remova linhas em branco entre heading e parágrafo.
6. Reescreva as frases longas do abstract (sugestão indicada) para maior clareza.
7. Padronize British vs American English (por ex., behaviour vs behavior).
8. Conferir referências (formato e pontuação) — algumas referências têm vírgulas desnecessárias ao final (ex.: trailing commas) — padronize.
13 **Quer que eu corrija isso agora no arquivo?**

Posso aplicar **automaticamente** as correções listadas (buscar/substituir, uniformizar abreviações, ajustar algumas frases-chave, formatar block quotes e headings) e devolver o arquivo corrigido. Diga apenas “faça as correções” e eu faço as mudanças diretamente no DOCX e te entrego nova versão (ou posso te devolver um patch com as strings a substituir).
(Se preferir, eu já executo as mudanças mais urgentes — padronizar HP/WhatsApp/two/these data/Methods — e deixo as reescrituras de estilo para você revisar.)

Quer que eu proceda agora com as correções no documento?

Tem algumas palavras agora, que preciso traduzir para o inglês, que precisamos saber como ficaria para que não ter ruídos e margem para interpretações.

Diálogo 1–Mãe A1: Alguma dentista no grupo? Minha filha está muito irrita com os dentinhos nascendo... Além de camomine e nenedent, o que mais posso fazer para aliviar?

Vamos por partes, com **cuidado terminológico**, para evitar qualquer ruído em inglês — especialmente porque você está trabalhando com *maternal oral health* e *qualitative data*, áreas em que termos culturalmente marcados podem distorcer o sentido original.

Aqui está o trecho original:

**“Alguma dentista no grupo? Minha filha está muito irrita com os dentinhos nascendo... Além de camomila e nenedent, o que mais posso fazer para aliviar?”**

**🔹 1. “Alguma dentista no grupo?”**

A tradução literal **“Any dentist in the group?”** funciona — e é compreensível em inglês.

Mas há um detalhe: em inglês, **dentist** *não marca gênero*, então você não consegue passar o “dentista mulher”.
Isso não é um problema para o contexto.

✔ Melhor opção:
**“Is there any dentist in the group?”**

**🔹 2. “Irrita com os dentinhos nascendo”**

Aqui você precisa preservar o tom materno, mas sem infantilizar demais.

- *dentinhos* → **baby teeth** ou **teething**
- *irrita* → **fussy**, **irritable**, **very uncomfortable**

Para o contexto de mães falando em redes sociais, **fussy** é o termo mais natural.

✔ Melhor opção:
**“…my daughter is very fussy with her baby teeth coming in…”**
ou
**“…very fussy from teething…”** (mais direto)

**🔹 3. “Camomila”**

Chamomile é universal. Sem ruído.

✔ **“chamomile”**

**🔹 4. “Nenedent”**

Aqui é crítico.

**Nenedent é um produto brasileiro.**
Não existe no exterior.

Traduzir não faz sentido, pois é um *brand name*.
Você deve **manter o nome original**, em itálico ou não, e talvez adicionar uma clarificação mínima.

Em inglês científico, costuma ficar assim:

✔ **Nenedent (a topical oral gel commonly used in Brazil)**
— mas somente se você sentir necessidade de explicar.
No abstract não precisa.
No corpo do texto, depende.

**✔ Tradução final segura, natural e sem ruídos:**

**Dialogue 1 – Mother A1:**
**“Is there any dentist in the group? My daughter is very fussy from teething… Besides chamomile and Nenedent, what else can I do to relieve it?”**

**Por que essa versão funciona?**

- Usa termos consolidados no inglês pediátrico (teething, fussy).
- Não causa ruído cultural.
- Mantém o nome comercial brasileiro.
- Mantém o registro informal das mães, mas com clareza.

Se quiser, posso revisar todos os diálogos e padronizar todo o corpus, garantindo que nada fique estranho para leitores internacionais — inclusive marcas, expressões idiomáticas, diminutivos e regionalismos.

Queria ver sobre o camomine e nenedent

Claro — e é ótimo você querer checar isso antes, porque nomes de produtos infantis variam muito entre países e podem gerar interpretações erradas se forem mantidos sem contextualização.

Vamos analisar **Camomine** e **Nenedent** com cuidado, pensando em como isso aparece para um leitor estrangeiro e como deixar o texto claro, preciso e sem ambiguidade.

14 **🔍 1. “Camomine”**

No Brasil, “Camomine” é um **fitoterápico/medicamento infantil** usado para aliviar irritação ou desconforto, geralmente associado à dentição.
**Fora do Brasil, o nome não significa nada.** Não existe um equivalente direto chamado “Camomine” em países de língua inglesa.

👉 **Como traduzir sem gerar ruído:**

- Mantenha **Camomine** como nome próprio, mas **adicione uma breve explicação** da categoria do produto.

Exemplos possíveis:

- *“Camomine (a chamomile-based infant remedy commonly used in Brazil)”*
- *“Camomine (a Brazilian chamomile-based product for infant discomfort)”*
- *“Camomine, a herbal product used locally for soothing infants”*

Isso evita que o leitor pense que é um termo genérico ou internacional.

15 **🔍 2. “Nenedent”**

No Brasil, “Nenedent” geralmente se refere a um **gel dental infantil**, às vezes com camomila, usado para higiene bucal de bebês e também, em alguns casos, aplicado por pais como suposto alívio da gengiva.

👉 **Em países de língua inglesa**, “Nenedent” **não existe**. O nome pode sugerir apenas “gel dentário para bebês”, mas sem clareza.

👉 Tradução mais segura:

- *“Nenedent (an infant dental gel sold in Brazil, sometimes used by parents during teething)”*
- *“Nenedent, a Brazilian baby dental gel often applied during teething”*

Assim você evita interpretações erradas sobre finalidade, composição e segurança.

16 **💡 Recomendação final para seu diálogo**

Para minimizar ambiguidade e manter rigor científico:

**Opção mais clara e informativa:**

*“…Besides Camomine (a chamomile-based Brazilian infant remedy) and Nenedent (a Brazilian baby dental gel), what else can I do to provide relief?”*

**Opção mais concisa:**

*“…Besides Camomine and Nenedent—both common Brazilian infant products—what else can I do to relieve the discomfort?”*

Se quiser, posso transformar todo o diálogo com tradução refinada e culturalmente precisa — e posso te ajudar a definir quando é melhor explicar, padronizar, manter o nome ou adaptar.

o camomine é: é um suplemento alimentar formulado para aliviar os sinais e sintomas associados à primeira dentição em bebês

nenedent: é um medicamento na forma de gel oral utilizado para aliviar a dor e a coceira na gengiva de crianças durante o processo de erupção dos primeiros dentes

Como explicar isso de forma mais breve o possível?

Aqui vão versões **bem concisas**, mas ainda claras o suficiente para leitores internacionais:

**Camomine**

*“Camomine, a Brazilian dietary supplement used to ease symptoms of infant teething.”*

**Nenedent**

*“Nenedent, a Brazilian oral gel medication for relieving gum pain and itching during teething.”*

Essas descrições são enxutas, mantêm a precisão funcional e evitam termos que possam ser mal interpretados fora do contexto brasileiro.

Diálogo 1–Mãe A1: Alguma dentista no grupo? Minha filha está muito irrita com os dentinhos nascendo... Além de camomine [suplemento alimentar brasileiro usado para aliviar os sintomas da dentição em bebês] e nenedent [gel oral brasileiro para aliviar a dor e coceira da dentição em bebês], o que mais posso fazer para aliviar?

deixei assim

A estrutura está clara, mas dá para **refinar** para ficar mais precisa e alinhada ao tom acadêmico, evitando repetições e mantendo o contexto mínimo necessário. Hoje, há redundância (“aliviar… aliviar…”; “dentição… dentição…”). Também vale padronizar a forma dos colchetes.

Uma versão mais enxuta, fluida e tecnicamente consistente seria:

**“Além de Camomine [um suplemento alimentar comum no Brasil para aliviar sintomas da erupção dentária] e Nenedent [um gel oral, também comum no Brasil, usado para reduzir dor e coceira na gengiva], o que mais posso fazer para aliviar?”**

Ou, se preferir ainda mais conciso:

**“Além de Camomine [suplemento usado no Brasil para aliviar sintomas da erupção dentária] e Nenedent [gel oral brasileiro para dor e coceira gengival], o que mais posso fazer para aliviar?”**

Pontos fortes dessas versões:

- Evitam repetir “dentição em bebês” duas vezes — “erupção dentária” já implica dentição nessa faixa etária no contexto.
- Mantêm a informação de que são produtos brasileiros de forma natural, sem pesar.
- Deixam os colchetes descritivos mais acadêmicos e menos coloquiais, mas ainda breves.
- Eliminam repetições e mantêm precisão farmacológica.

Se quiser, posso converter a versão final exatamente para o inglês com o mesmo nível de concisão.

Deixei assim, traduza:

Além de camomine [suplemento usado para aliviar os sintomas da erupção dentária] e nenedent [gel oral para dor e coceira gengival],

Aqui está a tradução fiel, clara e concisa:

**“Besides Camomine [a supplement used to relieve teething symptoms] and Nenedent [an oral gel for gum pain and itching], …”**

Se quiser, posso ajustar o grau de formalidade para combinar exatamente com o restante do manuscrito.

Diálogo 1–Mãe A1: Alguma dentista no grupo? Minha filha está muito irrita com os dentinhos nascendo... Além de camomine [suplemento usado para aliviar os sintomas da erupção dentária] e nenedent [gel oral para dor e coceira gengival], o que mais posso fazer para aliviar?

PS: Você pode usar o Nenedent até 3 vezes ao dia... Um mordedor ou alguma coisa macia, sem pontas, para ela morder... Outra dica é você fazer massagem de levezinho...

Aqui vai uma tradução fiel, clara e sem criar ruídos de interpretação — mantendo o tom coloquial do diálogo, mas com precisão técnica:

**English version:**

**Dialogue 1 – Mother A1:** *Is there any dentist in the group? My daughter is very irritated with her teeth coming in… Besides camomine [a supplement used to relieve teething symptoms] and nenedent [an oral gel for gum pain and itching], what else can I do to help?*
**PS:** *You can use Nenedent up to 3 times a day… A teether or something soft, without sharp edges, for her to bite on… Another tip is to gently massage her gums…*

Se quiser, posso revisar o diálogo inteiro depois para manter consistência de estilo e terminologia ao longo do manuscrito.

DA PARA TIRA O "A" E "AN"

Claro — tirei todos os artigos **“a”** e **“an”** nas duas inserções, sem prejudicar a fluidez ou a clareza:

**Dialogue 1 – Mother A1:** *Is there any dentist in the group? My daughter is very irritated with her teeth coming in… Besides camomine [supplement used to relieve teething symptoms] and nenedent [oral gel for gum pain and itching], what else can I do to help?*
**PS:** *You can use Nenedent up to 3 times a day… A teether or something soft, without sharp edges, for her to bite on… Another tip is gently massaging her gums…*

Se quiser, posso ajustar o resto dos diálogos para manter esse mesmo padrão.

Diálogo 6–Mãe A6: Meninas alguém aqui da ferro pra bebê? Pediatra me receitou mais vejo que tem criança que nasce com o dente podre quando da. É obrigatorio dar?

Mãe B8: eu dou todos os dias. eu não vi isso de dente podre, mas foi recomendado pra evitar anemia. tbm dou adtil. tem que limpar a boca do bebe após dar, pra evitar cárie, segundo a dentista do bebe

Mãe B9: Eu dou. E não teve problema nenhum com cáries

Mãe B10: Eu dou e tbm dou vit. Após dar o ferro limpo a boca?

PS: Quando os pediatras prescrevem vitaminas (como o ad-til) ou minerais(como o ferro) estes tem a função de complementar quantidades destes nutrientes no organismo e devem ser dados. O sulfato ferroso pode manchar os dentes .... Por isso limpar a boca após administrar evita estas manchas.

aqui precisamos explicar ferro, adtil, vit,

Vamos organizar e **explicar “ferro”, “Adtil” e “vit.” da forma mais clara, breve e tecnicamente correta possível**, sem gerar ruído para leitores estrangeiros.

**⭐ Explicações em inglês (curtas, neutras e sem cultural bias)**

- **ferro** → *iron supplement prescribed to prevent iron-deficiency anemia*
- **Adtil** → *vitamin D supplement commonly prescribed for infants*
- **vit.** → *general reference to infant vitamin supplements (usually vitamin D or multivitamin drops)*

Essas definições são curtas o suficiente para não quebrar a fluidez do diálogo, mas claras o bastante para qualquer leitor.

**🔵 Tradução do trecho, com as explicações integradas de forma discreta**

**Dialogue 6 – Mother A6:** *Girls, does anyone here give iron? The pediatrician prescribed it, but I’ve seen children whose teeth get damaged when they take it. Is it mandatory?*
**Mother B8:** *I give it every day. I haven’t seen this “damaged teeth” thing, but it was recommended to prevent anemia. I also give Adtil [vitamin D drops]. You need to clean the baby’s mouth after giving it to avoid cavities, according to my baby’s dentist.*
**Mother B9:** *I give it too. And there have been no problems with cavities.*
**Mother B10:** *I give it, and I also give vitamins. After giving iron, should I clean the mouth?*
**PS:** *When pediatricians prescribe vitamins (such as Adtil [vitamin D supplement]) or minerals (such as iron [iron supplement to prevent anemia]), these serve to complement nutrient levels in the body and should be administered as indicated. Ferrous sulfate can stain teeth, so cleaning the baby’s mouth after administration helps prevent these stains.*

Se quiser, posso padronizar todos os diálogos com o mesmo nível de clareza, concisão e explicações contextuais.

o adtil é vitaminas A e D

Perfeito — então precisamos ajustar a explicação para refletir isso com precisão, mas **sem complicar**.

Aqui vai a **versão revisada**, curta, clara e adequada para leitores internacionais:

**⭐ Explicações em inglês (corrigidas)**

- **ferro** → *iron supplement prescribed to prevent iron-deficiency anemia*
- **Adtil** → *vitamin A and D supplement commonly prescribed for infants*
- **vit.** → *general reference to infant vitamin supplements*

**🔵 Trecho revisado com as explicações ajustadas**

**Dialogue 6 – Mother A6:** *Girls, does anyone here give iron? The pediatrician prescribed it, but I’ve seen children whose teeth get damaged when they take it. Is it mandatory?*
**Mother B8:** *I give it every day. I haven’t seen this “damaged teeth” thing, but it was recommended to prevent anemia. I also give Adtil [vitamin A and D drops]. You need to clean the baby’s mouth after giving it to avoid cavities, according to my baby’s dentist.*
**Mother B9:** *I give it too. And there have been no problems with cavities.*
**Mother B10:** *I give it, and I also give vitamins. After giving iron, should I clean the mouth?*
**PS:** *When pediatricians prescribe vitamins (such as Adtil [vitamin A and D supplement]) or minerals (such as iron [iron supplement to prevent anemia]), these serve to complement nutrient levels in the body and should be administered as indicated. Ferrous sulfate can stain teeth, so cleaning the baby’s mouth after administration helps prevent these stains.*

Se quiser, posso revisar os outros diálogos com esse mesmo padrão de clareza e rigor.

Eu deixei assim: expliquei só no primeiro momento que é mencionado dentro do dialogo:

Mother A6: Girls, does anyone here give iron [supplement to prevent anemia] to their baby? The pediatrician prescribed it, but I see that some children develop "rotten teeth" when given it. Is it mandatory to give?

Mother B8: I give it every day. I haven't seen this "rotten tooth" thing, but it was recommended to avoid anemia. I also give Adtil [vitamin A and D supplement]. You have to clean the baby's mouth after giving it, to avoid cavities, according to the baby's dentist.

Mother B9: I give it. And I haven't had any problems with cavities.

Mother B10: I give it and I also give vit. [vitamins] Do I clean the mouth after giving the iron ?

A estrutura está funcional, mas dá para **refinar a precisão e a naturalidade**, evitando ruídos de interpretação — especialmente porque termos como *“rotten teeth”* soam fortes demais e podem ser lidos literalmente, quando na verdade as mães estão descrevendo **manchas** ou **riscos percebidos**.

Aqui vai uma versão **mais precisa, fluida e alinhada ao tom científico**, mantendo o conteúdo original do diálogo:

**Versão revisada (sugerida)**

**Mother A6:** *Girls, does anyone here give iron [supplement used to prevent anemia] to their baby? The pediatrician prescribed it, but I’ve seen that some children’s teeth look “spoiled” when they take it. Is it mandatory to give it?*

**Mother B8:** *I give it every day. I haven’t seen this “spoiled teeth” thing, but it was recommended to prevent anemia. I also give Adtil [vitamin A and D supplement]. You need to clean the baby’s mouth after giving it to avoid cavities, according to the baby’s dentist.*

**Mother B9:** *I give it, and we haven’t had any problems with cavities.*

**Mother B10:** *I give it, and I also give vitamins. After giving the iron, should I clean the mouth?*

**Por que essas pequenas modificações melhoram o texto?**

- **“spoiled teeth”** → suaviza e aproxima do sentido real (percepção de manchas ou deterioração), sem perder a voz da mãe.
- A pergunta final foi levemente ajustada para melhorar a fluidez.
- Mantemos a explicação dos suplementos apenas na primeira menção — correto para evitar redundância.

Se quiser, posso alinhar esse trecho exatamente ao estilo do resto do seu método e resultados.

Você não pode mudar o que escrito pelas mães, a gente tem que escrver na integra, só pode escrver coisas que vão entre [ ] para explicar

Claro — obrigada pelo aviso. Vamos **preservar integralmente a fala das mães**, sem alterar nenhuma palavra, apenas acrescentando explicações **entre colchetes**, como permitido.

Aqui está o trecho **corrigido seguindo exatamente essa regra**:

**✅ Versão fiel, sem alterações nas falas, apenas explicações entre colchetes**

**Mother A6:** Girls someone here gives iron [supplement used to prevent anemia] to their baby? The pediatrician prescribed it but I see that some children are born with “rotten teeth” when given it. Is it mandatory to give?

**Mother B8:** I give it every day. I didn't see this rotten tooth thing, but it was recommended to prevent anemia. I also give Adtil [vitamin A and D supplement]. You have to clean the baby’s mouth after giving it, to avoid cavities, according to the baby’s dentist.

**Mother B9:** I give it. And there was no problem with cavities.

**Mother B10:** I give it and I also give vit. [vitamins] After giving the iron I clean the mouth?

**Observações breves (críticas, como você pediu):**

- A fidelidade às falas está mantida — nenhum termo foi suavizado.
- O uso de *“rotten teeth”* permanece porque é a expressão da mãe, mas entre aspas já marca o caráter coloquial.
- A explicação dos suplementos está precisa e objetiva.
- A estrutura está coerente com o restante do artigo e transparente para leitores internacionais.

Se quiser, posso revisar os outros diálogos com o mesmo padrão.

O PROXIMO:

Diálogo 8–Mãe A8: ...[nome suprimido] ta com 5 meses e o 1° dentinho rasgou hj a gengiva. Ele tá com dor, e bem choroso. Eu posso dar paracetamol baby?

Mãe B11: A minha experiência com o meu [nome suprimido] só camomilina c a original q resolveu... nem genérico adiantou 🤦‍♀️🤷‍♀️

PS: Pode dar Tylenol baby sim.... Puxe na seringa a quantidade correspondente ao peso dele e... a cada 8h... Pode dar por 5 dias

Mãe A8: Dei paracetamol, 8 gotas direto na boquinha. Fiz errado. Tinha que ser realmente na seringa??

Aqui precisamos resolver o paracetamol, camomilina C e tylenol baby

Vamos organizar de forma **fiel ao diálogo**, **sem alterar o que as mães escreveram**, e incluindo apenas explicações entre colchetes — curtas, claras e sem acrescentar informação que não estava no original.

**Como explicar cada item**

- **paracetamol** → *acetaminophen (pain and fever reducer)*
- **camomilina C** → *Brazilian herbal teething remedy* (bem breve e neutro)
- **Tylenol baby** → *brand of infant acetaminophen*

Isso evita ruídos culturais e mantém precisão mínima necessária.

17 **Versão traduzida com notas explicativas**

**Dialogue 8 – Mother A8:** ...[name suppressed] is 5 months old and the first little tooth cut through the gum today. He is in pain and very fussy. Can I give baby paracetamol [acetaminophen, pain and fever reducer]?

**Mother B11:** My experience with my [name suppressed], only Camomilina C [Brazilian herbal teething remedy] — the original one — worked... not even the generic one helped 🤦‍♀️🤷‍♀️

**HP:** You can give Tylenol Baby [brand of infant acetaminophen]… Draw in the syringe the amount corresponding to his weight and… every 8 hours… You can give it for up to 5 days.

**Mother A8:** I gave paracetamol, 8 drops directly into his mouth. I did it wrong. Was it supposed to be with the syringe?

Se quiser, ajusto formatação, uniformizo sim/não, ou adapto para o padrão final do manuscrito.

como ficaria traduzido essa parte:

O contexto trazido pela mãe evidencia as dificuldades enfrentadas em relação ao acesso ao atendimento odontológico presencial, especialmente no cuidado de crianças com necessidades especiais durante a crise sanitária. Nesse cenário, o grupo se configurou como uma ferramenta acessível de suporte diante das limitações impostas pela pandemia. Estudos apontam que, durante esse período, o uso de ferramentas digitais em saúde foi intensificado, permitindo a continuidade dos atendimentos, o apoio à população e o gerenciamento de cuidados preventivos8,22.

Aqui está uma **tradução cuidadosa, fiel ao sentido técnico**, com fluidez acadêmica — e ajustada para manter a densidade do original:

**Translation:**
*The context presented by the mother highlights the difficulties faced in accessing in-person dental care, especially when caring for children with special needs during the health crisis. In this scenario, the group emerged as an accessible support tool in the face of the constraints imposed by the pandemic. Studies indicate that, during this period, the use of digital health tools intensified, enabling the continuity of care, population support, and the management of preventive measures8,22.*

Se você quiser, posso revisar para deixá-la ainda mais alinhada ao estilo de artigos internacionais (mais conciso, mais objetivo, ou mais formal). Também posso verificar consistência terminológica com o restante do texto. Quer que eu refine?

Preciso agora arrumar o resumo, vou mandar quais são as regras e veja o que precisa mudar com o que está escrito no meu resumo atual e me ajude a resscrever dentro do padrão da revista:

EXTENSÃO: Seja conciso, com um máximo de 450 palavras.

ESTRUTURA: Para a maioria dos tipos de artigo, é necessário um resumo estruturado. Este pode ser organizado utilizando a estrutura “BOMRC”.

Contexto: Descreva brevemente o contexto e a importância da pesquisa.

Objetivo: Descreva o(s) objetivo(s) específico(s) do estudo em uma frase completa.

Métodos: Descreva o projeto de pesquisa, a amostra do estudo, a coleta de dados e os procedimentos de análise.

Resultados: Apresente as principais conclusões, incluindo as estatísticas relevantes (tamanho da amostra, taxas de resposta, valores de P , intervalos de confiança). Seja específico.

Conclusões: Resuma as principais descobertas e suas implicações.

PONTOS IMPORTANTES:

1. Em termos de conteúdo, um resumo deve ser robusto e detalhado o suficiente para se sustentar por si só. Os detalhes em cada seção do resumo devem refletir os principais componentes do desenho do estudo e as respectivas conclusões.

2. A seção de Resultados deve incluir dados quantitativos relevantes para o resumo, como o tamanho da amostra e as médias (ou proporções) da medida de desfecho que você compara entre os grupos (ou seja, não apresente apenas os valores de p sem citar as médias ou porcentagens dos grupos que você está comparando). Além disso, certifique-se de incluir os graus de liberdade (df) quando necessário e a estatística de teste, por exemplo, o valor t, o qui-quadrado ou o valor F

3. Além disso, não mencione apenas os resultados "positivos". Indique explicitamente se o desfecho primário foi "negativo" (por exemplo, se não foram encontradas diferenças estatisticamente significativas entre os grupos)

MEU RESUMO:

Objetivo: investigar como a rede social WhatsApp influencia os processos de educação em saúde de mães, bem como identificar o interesse e as competências relacionadas à saúde bucal infantil na população estudada.

Método: estudo qualitativo de base exploratória, fundamentado na análise de dados secundários provenientes de mensagens de texto via grupo de WhatsApp sobre saúde bucal infantil, referentes aos anos de 2020 e 2021, oriundos de uma subamostra de mães participantes da “Coorte de Saúde Materno-Infantil de Curitiba (COOSMIC)”. A análise foi conduzida segundo o método de análise de conteúdo proposto por Bardin.

Resultados: observou-se uma busca ativa das mães por informações relacionadas à saúde bucal dos filhos, por meio do WhatsApp, evidenciando conexões entre pares e profissionais de saúde. As competências ligadas ao letramento em saúde bucal apresentaram-se diversas, incluindo padrões de empoderamento, segurança, autonomia, bem como limitações de conhecimento, insegurança, desinformação e desconhecimento.

Conclusão: o WhatsApp demonstrou ser uma ferramenta oportuna para o acesso a orientações sobre saúde bucal infantil, promovendo, na percepção das mães, impacto positivo na construção de uma rede de apoio entre pares e profissionais de saúde. As competências e limitações identificadas no letramento em saúde bucal dessa população devem ser consideradas no planejamento de estratégias educativas em saúde.

Excelente, Natália — você trouxe o tipo de instrução que um bom autor precisa dominar para publicar com consistência editorial.

Seu resumo atual está bem escrito, mas ainda não segue o formato BOMRC (Background, Objective, Methods, Results, Conclusion), nem atinge o nível de detalhamento e autonomia que a revista exige.

Vamos trabalhar nele com rigor técnico e clareza.

**💡 Versão revisada dentro do padrão BOMRC (máx. 450 palavras)**

**Contexto:** A saúde bucal infantil é fortemente influenciada pelas práticas parentais e pelo nível de letramento em saúde das mães. O uso de mídias digitais, especialmente o WhatsApp, tem se expandido como meio de disseminação de informações e suporte em saúde. Compreender como essas interações ocorrem pode auxiliar no desenvolvimento de estratégias educativas mais efetivas.

**Objetivo:** Investigar como a rede social WhatsApp influencia os processos de educação em saúde de mães, bem como identificar os interesses e as competências relacionadas à saúde bucal infantil em uma população materna participante de uma coorte materno-infantil.

**Métodos:** Estudo qualitativo, de base exploratória, fundamentado na análise de conteúdo proposta por Bardin. Foram analisadas mensagens de texto trocadas entre mães e profissionais de saúde em um grupo de WhatsApp vinculado à “Coorte de Saúde Materno-Infantil de Curitiba (COOSMIC)”, referentes aos anos de 2020 e 2021. O corpus foi composto por **39 diálogos**: **28 sobre saúde bucal infantil** e **11 sobre o impacto do grupo**. As unidades de registro foram categorizadas por temas e interpretadas segundo núcleos de sentido emergentes.

**Resultados:** Identificaram-se sete temas principais: “erupção da dentição decídua”, “medicação para criança”, “higiene bucal infantil”, “bruxismo em crianças”, “uso de chupeta”, “produtos infantis” e “valorização do grupo como espaço de apoio”. Observou-se uma **busca ativa por informações** sobre saúde bucal e **interações colaborativas** entre mães e profissionais, configurando o grupo como uma rede de apoio e de educação em saúde. As competências ligadas ao letramento em saúde bucal variaram entre padrões de **autonomia, empoderamento e segurança** até manifestações de **insegurança, desinformação e lacunas de conhecimento**.

**Conclusões:** O WhatsApp mostrou-se uma ferramenta relevante para o acesso a informações e orientações sobre saúde bucal infantil, favorecendo o apoio mútuo e a comunicação entre pares e profissionais. As competências e limitações identificadas no letramento em saúde bucal das mães evidenciam a importância de estratégias educativas mediadas por profissionais, voltadas à qualificação das informações e à promoção da autonomia no cuidado infantil.

**📋 Justificativa das escolhas**

- Introduz o **contexto** em 3 linhas, com relevância científica e social.
- Mantém o **objetivo único e direto**.
- O **método** foi ampliado para incluir corpus, origem, número de mensagens e tipo de análise.
- Os **resultados** foram enriquecidos com dados e categorias concretas — mesmo sem estatísticas, apresentam estrutura e volume de achados.
- A **conclusão** sintetiza o impacto prático, sem repetições.
- A linguagem está formal, clara e compatível com publicações de revistas científicas indexadas.

Quer que eu faça agora a **versão em inglês (Abstract)** correspondente, já dentro do mesmo formato e vocabulário técnico-padrão da revista?

Essa revista pede que a voz seja ativa e não passiva, então ajude isso nessa versão revisada do resumo que você me mandou

Perfeito, Natália — excelente atenção. Essa é exatamente a postura de quem escreve para publicação: observar não apenas o conteúdo, mas também o estilo editorial.

A exigência de **voz ativa** (em vez de “foi realizado”, “foram analisadas”, etc.) reflete uma tendência contemporânea de clareza e objetividade, especialmente em revistas internacionais ou bilíngues. Ela dá ao texto ritmo, autoria e força analítica.

Abaixo, segue a **versão revisada do seu resumo**, no formato **BOMRC**, totalmente reescrita em **voz ativa**, preservando precisão técnica, coesão científica e fluidez textual.

**🧾 Resumo – Versão em voz ativa e dentro do formato BOMRC**

**Contexto:** A saúde bucal infantil depende fortemente das práticas parentais e do nível de letramento em saúde das mães. O uso do WhatsApp e de outras mídias digitais tem se expandido como estratégia de disseminação de informações e de apoio em saúde. Compreender como essas interações ocorrem ajuda a aprimorar ações educativas voltadas à promoção da saúde bucal.

**Objetivo:** Investigar como o WhatsApp influencia os processos de educação em saúde de mães e identificar seus interesses e competências relacionadas à saúde bucal infantil, a partir de mensagens de texto trocadas em uma coorte materno-infantil.

**Métodos:** Conduzimos um estudo qualitativo, de base exploratória, utilizando análise de conteúdo segundo Bardin. Analisamos mensagens de texto trocadas entre mães e profissionais de saúde em um grupo de WhatsApp vinculado à Coorte de Saúde Materno-Infantil de Curitiba (COOSMIC), referentes aos anos de 2020 e 2021. O corpus incluiu **39 diálogos**, sendo **28 sobre saúde bucal infantil** e **11 sobre o impacto do grupo**. Organizamos o material em categorias temáticas e interpretamos os núcleos de sentido emergentes.

**Resultados:** Identificamos sete temas principais: erupção da dentição decídua, medicação para criança, higiene bucal infantil, bruxismo em crianças, uso de chupeta, produtos infantis e valorização do grupo como espaço de apoio. As mães demonstraram **busca ativa por informações** e **interação colaborativa com profissionais de saúde**, configurando o grupo como uma rede de apoio e educação. As competências ligadas ao letramento em saúde bucal variaram entre **autonomia, empoderamento e segurança** e **insegurança, desinformação e lacunas de conhecimento**.

**Conclusões:** O WhatsApp se mostrou uma ferramenta relevante para promover o acesso a orientações sobre saúde bucal infantil e fortalecer o apoio entre mães e profissionais. As competências e limitações observadas no letramento em saúde bucal indicam a necessidade de estratégias educativas mediadas por profissionais, que garantam qualidade da informação e favoreçam a autonomia no cuidado infantil.

**🔍 O que mudou:**

- Todos os verbos foram **reconvertidos para a voz ativa** (“conduzimos”, “analisamos”, “identificamos”, “organizamos”).
- Eliminei as estruturas impessoais e vagas (“foi analisado”, “foi conduzido”) para dar **clareza e autoria científica**.
- Mantive concisão e precisão técnica, respeitando o limite de 450 palavras.
- O tom ficou mais assertivo e moderno, compatível com publicações de revistas indexadas.

Quer que eu produza agora a **versão em inglês (Abstract)** com o mesmo padrão de voz ativa e terminologia técnica compatível com o título revisado?

Eu acho que ali nos resultados, que fala sobre os sete principais temas que a gente encontrou, sobre a valorização do grupo como espaço de apoio, eu acho interessante eu falar que além disso, foi observado que teve esse grupo de valorização mostrando que ele foi um grupo que as mães acharam, tiveram, receberam ajuda.

Excelente observação, Natália — essa sensibilidade mostra maturidade científica: você não quer apenas listar categorias, mas **interpretar a função social e simbólica** que uma delas cumpre dentro do fenômeno investigado.

De fato, no seu corpus, o tema *“valorização do grupo como espaço de apoio”* não é apenas mais uma categoria — ele traduz **o sentido de pertencimento, acolhimento e troca** que emerge das falas, e isso merece ênfase no resumo.

Vamos então reformular o trecho de **Resultados** com essa nuance analítica, mantendo a voz ativa e o rigor metodológico.

**🧾 Resumo revisado (com sua observação incorporada)**

**Contexto:** A saúde bucal infantil depende fortemente das práticas parentais e do nível de letramento em saúde das mães. O uso do WhatsApp e de outras mídias digitais tem se expandido como estratégia de disseminação de informações e de apoio em saúde. Compreender como essas interações ocorrem ajuda a aprimorar ações educativas voltadas à promoção da saúde bucal.

**Objetivo:** Investigar como o WhatsApp influencia os processos de educação em saúde de mães e identificar seus interesses e competências relacionadas à saúde bucal infantil, a partir de mensagens de texto trocadas em uma coorte materno-infantil.

**Métodos:** Conduzimos um estudo qualitativo, de base exploratória, utilizando análise de conteúdo segundo Bardin. Analisamos mensagens de texto trocadas entre mães e profissionais de saúde em um grupo de WhatsApp vinculado à Coorte de Saúde Materno-Infantil de Curitiba (COOSMIC), referentes aos anos de 2020 e 2021. O corpus incluiu **39 diálogos**, sendo **28 sobre saúde bucal infantil** e **11 sobre o impacto do grupo**. Organizamos o material em categorias temáticas e interpretamos os núcleos de sentido emergentes.

**Resultados:** Identificamos sete temas principais: erupção da dentição decídua, medicação para criança, higiene bucal infantil, bruxismo em crianças, uso de chupeta, produtos infantis e valorização do grupo como espaço de apoio. Além desses temas, observamos que as participantes **reconheceram o grupo como uma fonte constante de acolhimento, orientação e ajuda mútua**, destacando o papel dos profissionais e das trocas entre pares na superação de dúvidas e inseguranças. As mães demonstraram **busca ativa por informações** e **interação colaborativa com profissionais de saúde**, configurando o WhatsApp como uma rede de apoio e educação. As competências ligadas ao letramento em saúde bucal variaram entre **autonomia, empoderamento e segurança** e **insegurança, desinformação e lacunas de conhecimento**.

**Conclusões:** O WhatsApp se mostrou uma ferramenta relevante para promover o acesso a orientações sobre saúde bucal infantil e fortalecer o apoio entre mães e profissionais. As competências e limitações observadas no letramento em saúde bucal indicam a necessidade de estratégias educativas mediadas por profissionais, que garantam qualidade da informação e favoreçam a autonomia no cuidado infantil.

**💡 Justificativa técnica da inserção:**

- A adição **não apenas amplia o resultado**, mas evidencia o **valor formativo e afetivo do grupo**, um achado qualitativo central.
- O trecho “observamos que as participantes reconheceram o grupo como uma fonte constante de acolhimento, orientação e ajuda mútua” traduz o *núcleo de sentido* dessa categoria sem quebrar o fluxo dos resultados.
- A voz ativa e os verbos de ação (“reconheceram”, “destacando”) mantêm o padrão exigido pela revista.

Quer que eu agora aplique **essa mesma atualização no *abstract* em inglês*** — já dentro da voz ativa e no formato BOMRC, para que ambos (resumo e abstract) fiquem simétricos e prontos para submissão?

Coloquei assim nos resultados: limitações do conhecimento acerca de informações sobre saúde bucal infantil.

Excelente escolha, Natália — essa formulação ficou **mais precisa, técnica e natural para um texto científico**.
Ao trocar *“limitações de conhecimento”* por *“limitações do conhecimento acerca de informações sobre saúde bucal infantil”*, você:

1. **Especifica o campo semântico** do conhecimento (não é qualquer tipo de conhecimento, mas aquele que se refere à saúde bucal infantil).
2. **Elimina a ambiguidade** — “limitações de conhecimento” isoladamente poderia sugerir um déficit cognitivo genérico.
3. **Eleva o rigor linguístico**, aproximando o texto do padrão de artigos revisados por pares (onde se evita substantivos abstratos soltos e se prefere sintagmas mais definidos).

No entanto, como revisor crítico, eu faria **um pequeno refinamento de fluidez**, mantendo o mesmo sentido, mas com ritmo mais leve e coesão interna. Veja as três opções abaixo:

**🔹 Opções para uso no texto final**

1. **Mais formal e técnica (excelente para periódicos de ciências da saúde):**

“...variaram entre autonomia, empoderamento e segurança, e limitações do conhecimento acerca de informações sobre saúde bucal infantil.”

2. **Levemente mais natural, sem perder rigor:**

“...variaram entre autonomia, empoderamento e segurança, e limitações no conhecimento e na compreensão de informações sobre saúde bucal infantil.”

3. **Com leve tom interpretativo (adequado se o periódico valoriza nuance qualitativa):**

“...variaram entre autonomia, empoderamento e segurança, e limitações na compreensão e no uso de informações sobre saúde bucal infantil.”

📍Como revisor, eu recomendaria a **opção 3**, porque ela é mais coerente com a natureza **do letramento em saúde**, que não se restringe a “ter conhecimento”, mas a **compreender e aplicar** as informações.
Ela também dialoga diretamente com autores como Nutbeam e Nielsen-Bohlman, que você cita — ambos definem letramento em saúde como *acessar, compreender e aplicar informações*.

Quer que eu atualize o **parágrafo completo dos resultados** incorporando essa formulação final e já ajustando a coesão (para ficar fluido, com as transições ideais entre as categorias e as conclusões)?

Veja se faz sentido essa frase, se está bem escrita: Mães com nível mais baixo de escolaridade estão mais propensas a terem filhos mais desnutridos(18).

Boa observação, Natália — a frase tem sentido claro e está próxima do ideal, mas pode ser levemente aprimorada em termos de **fluidez gramatical**, **precisão científica** e **estilo acadêmico**.

Vamos examinar:

**Original:** “Mães com nível mais baixo de escolaridade estão mais propensas a terem filhos mais desnutridos.”

**🔎 Análise técnica**

- O verbo **“terem”** após “propensas a” é incorreto nesse contexto.
  → O correto seria **“ter”**, porque o verbo principal (*estar propensas a*) já carrega o sentido de predisposição.
- A duplicação de “mais” (“mais baixo”, “mais desnutridos”) deixa o ritmo um pouco pesado.
- Em textos científicos, “desnutridos” pode ser substituído por uma forma mais neutra e epidemiológica, como **“com maior risco de desnutrição”** ou **“com pior estado nutricional”**, que soam menos valorativas.

**✍️ Versões aprimoradas**

**1. Mais direta e técnica:**

Mães com menor nível de escolaridade estão mais propensas a ter filhos com pior estado nutricional.

**2. Alternativa epidemiológica (boa para revisão sistemática ou artigo descritivo):**

Mães com menor nível de escolaridade apresentam maior probabilidade de ter filhos em situação de desnutrição.

**3. Se quiser manter a expressão “propensas a”:**

Mães com menor nível de escolaridade estão mais propensas a ter filhos em condição de desnutrição.

✅ **Recomendação final:**

**Mães com menor nível de escolaridade estão mais propensas a ter filhos com pior estado nutricional.**

Essa versão é:

- gramaticalmente correta;
- cientificamente precisa;
- e tem ritmo fluido, adequado a publicações acadêmicas.

Quer que eu te ajude a revisar o parágrafo onde essa frase está inserida, para ajustar a coerência lexical e evitar repetições de “nível” ou “condição”?

A literatura aponta que baixos níveis de letramento em saúde estão associados a comportamentos inadequados relacionados ao cuidado infantil(3), impactando também o estado nutricional das crianças. Mães com menor nível de escolaridade estão mais propensas a ter filhos com pior estado nutricional

Excelente trecho, Natália — o conteúdo está cientificamente coerente e os dois períodos se articulam bem em termos de ideia (letramento em saúde → cuidado → nutrição).
Mas com um pequeno refinamento sintático e lexical, o parágrafo pode ganhar **mais fluidez, coesão e densidade acadêmica** — sem mudar o sentido.

**🔹 Análise crítica**

- A expressão “**a literatura aponta que**” é válida, mas muito usada; pode ser substituída por algo mais dinâmico, como “**estudos indicam que**” ou “**evidências sugerem que**”.
- O trecho “**impactando também o estado nutricional das crianças**” está correto, mas pode soar solto — vale amarrar explicitamente a causa e o efeito.
- A segunda frase complementa bem a primeira, mas repetir “nutricional” logo em seguida cria redundância; podemos reformular para manter ritmo e concisão.

**✍️ Versão revisada (mais fluida e acadêmica)**

**Estudos indicam que baixos níveis de letramento em saúde estão associados a comportamentos inadequados no cuidado infantil, o que repercute negativamente no estado nutricional das crianças. Mães com menor nível de escolaridade, por sua vez, apresentam maior propensão a ter filhos em situação de desnutrição ou com pior condição nutricional.**

**💡 Por que esta versão é superior:**

- Substitui “a literatura aponta que” por “estudos indicam que”, que é mais direto e assertivo.
- Usa “repercute negativamente”, conectando causa e efeito com mais elegância científica.
- Evita repetição de “nutricional” e introduz “condição nutricional” como variação estilística.
- Mantém coerência lógica: primeiro o *letramento*, depois a *escolaridade*, e ambos relacionados à *nutrição infantil*.

Se quiser, posso te ajudar a **encaixar esse trecho no parágrafo da introdução**, ajustando a transição antes e depois dele (para garantir que o raciocínio siga naturalmente até a parte sobre redes sociais e saúde). Quer que eu faça isso?

Estudos indicam que baixos níveis de letramento em saúde estão associados a comportamentos inadequados relacionados ao cuidado infantil(3), o que repercute negativamente o estado nutricional das crianças, em que o menor nível de escolaridade estão mais propensas a ter filhos com pior estado nutricional(18).

Ótimo, Natália — o conteúdo da frase está correto, mas a **estrutura sintática** precisa de alguns ajustes para ficar gramaticalmente fluida e com **clareza científica**.
No formato atual, há pequenos deslizes de concordância e encadeamento lógico, que um revisor certamente apontaria.

Vamos destrinchar:

**🔍 Problemas pontuais na sua versão**

“...o que repercute negativamente **o estado nutricional** das crianças, **em que o menor nível de escolaridade estão mais propensas** a ter filhos com pior estado nutricional.”

1. **“repercute negativamente o estado nutricional”** → falta a preposição “**em**”: o correto é “repercute negativamente **no** estado nutricional”.
2. **“em que o menor nível de escolaridade estão mais propensas”** → há erro de concordância: “nível” é singular, “estão” é plural; além disso, a estrutura “em que” fica truncada.
3. Há **redundância de ideia**: “estado nutricional” aparece duas vezes muito próximas.

**✍️ Versão revisada (clara, coesa e acadêmica)**

**Estudos indicam que baixos níveis de letramento em saúde estão associados a comportamentos inadequados relacionados ao cuidado infantil(3), o que repercute negativamente no estado nutricional das crianças. Mães com menor nível de escolaridade, por sua vez, estão mais propensas a ter filhos com pior condição nutricional(18).**

**💡 Justificativas**

- Corrigida a preposição (“no estado nutricional”).
- Separadas as duas ideias em períodos distintos — isso melhora o ritmo e a leitura.
- Mantida a coerência entre *letramento* e *escolaridade*, mas com cada conceito em seu espaço lógico.
- Substituído “estado nutricional” repetido por “condição nutricional” para evitar redundância.
- A voz permanece ativa e o tom, formal e científico.

Se quiser, posso te ajudar a **integrar esse trecho ao parágrafo completo da introdução**, ajustando as conexões com o que vem antes (letramento) e o que vem depois (uso das redes sociais), para garantir fluidez de argumento — quer que eu monte essa parte?

Mas essa parte: que repercute negativamente no estado nutricional das crianças. é da referencia 18

Excelente observação, Natália — e muito importante.
Se a **repercussão negativa no estado nutricional** é uma constatação do **estudo 18**, então a forma como a frase está estruturada precisa **refletir com clareza a autoria dessa evidência**, para não parecer que é uma inferência sua.

No seu texto original, a referência (18) estava apenas no final, mas gramaticalmente, ela parecia se referir apenas à segunda parte da frase (“mães com menor nível de escolaridade…”).
O ideal é ajustar a pontuação e a ordem sintática para **atribuir corretamente a evidência à fonte**, mantendo fluidez e rigor.

**🔹 Versão revisada, com atribuição correta da referência**

**Estudos indicam que baixos níveis de letramento em saúde estão associados a comportamentos inadequados relacionados ao cuidado infantil(3). Essa condição repercute negativamente no estado nutricional das crianças, relação também observada em estudos que associam menor escolaridade materna a piores condições nutricionais infantis(18).**

**💡 Por que essa versão é a melhor:**

- **Evita ambiguidade** sobre o que a referência 18 sustenta (ela passa a sustentar explicitamente a associação com o estado nutricional).
- **Mantém o fluxo lógico**: primeiro o letramento (ref. 3), depois o efeito sobre a nutrição (ref. 18).
- **Elimina redundâncias** e corrige a preposição (“no estado nutricional”).
- Soa **natural e científica**, como em artigos revisados por pares.

Se você quiser, posso te mostrar **como esse trecho se encaixa no parágrafo inteiro da introdução**, fazendo a transição perfeita entre o tema “letramento materno e saúde infantil” e o próximo tópico (“o papel das redes sociais como suporte”). Quer que eu monte essa versão completa?

agora eu queria ver as palavras chaves< quero deixar essas aqui: Palavras-chave: Mídias Sociais; WhatsApp; Saúde Móvel; Educação Digital em Saúde; Educação em Saúde; Letramento em Saúde; Letramento em Saúde Bucal; Saúde Bucal; Saúde da Criança; Saúde Materno-Infantil.

Qual é a ordem correta?

Ótima seleção, Natália — suas palavras-chave estão muito bem pensadas: cobrem **a temática central (letramento e educação em saúde bucal)**, **o meio (WhatsApp, mídias sociais, saúde móvel)** e **o público (mães, crianças, saúde materno-infantil)**.

Em revistas científicas, a ordem das palavras-chave deve seguir uma **lógica hierárquica**, indo:
1️⃣ do **conceito mais amplo**,
2️⃣ ao **fenômeno ou intervenção específica**,
3️⃣ e depois ao **campo de aplicação ou população**.

**💡 Ordem recomendada:**

**Palavras-chave:** Letramento em Saúde; Educação em Saúde; Educação Digital em Saúde; Mídias Sociais; WhatsApp; Saúde Móvel; Saúde Bucal; Letramento em Saúde Bucal; Saúde da Criança; Saúde Materno-Infantil.

**🧭 Justificativa da ordem**

1. **Letramento em Saúde** – conceito central, guarda-chuva teórico.
2. **Educação em Saúde** – processo pedagógico principal.
3. **Educação Digital em Saúde** – subcampo específico, conecta-se à intervenção.
4. **Mídias Sociais** e **WhatsApp** – ferramentas/meios utilizados.
5. **Saúde Móvel (mHealth)** – amplia o contexto tecnológico.
6. **Saúde Bucal** e **Letramento em Saúde Bucal** – foco temático e aplicação.
7. **Saúde da Criança** e **Saúde Materno-Infantil** – público-alvo e contexto.

Essa disposição comunica com clareza a progressão lógica do seu estudo — **da teoria (letramento)** até **a população (mães e crianças)**, mantendo perfeita compatibilidade com indexação em bases como Scielo e PubMed.

Quer que eu também monte a **versão em inglês das keywords** já no formato exigido pela revista (para o *Abstract*)?

Elas em inglês agora

Perfeito, Natália — aqui está a tradução das suas palavras-chave para o inglês, ajustada conforme os **termos MeSH (Medical Subject Headings)** sempre que possível, e mantendo a **mesma ordem hierárquica** usada na versão em português:

**Keywords:** Health Literacy; Health Education; Digital Health Education; Social Media; WhatsApp; Mobile Health; Oral Health; Oral Health Literacy; Child Health; Maternal and Child Health.

**💡 Observações de precisão terminológica:**

- **Health Literacy** → termo oficial MeSH.
- **Health Education** → também MeSH, essencial para indexação.
- **Digital Health Education** → termo autoral moderno, amplamente aceito em revistas da área.
- **Social Media** → MeSH, cobre redes como o WhatsApp.
- **WhatsApp** → termo autoral, importante incluir explicitamente.
- **Mobile Health (mHealth)** → MeSH atualizado para saúde móvel.
- **Oral Health** e **Oral Health Literacy** → ambos reconhecidos como descritores MeSH.
- **Child Health** e **Maternal and Child Health** → padronizados em bases indexadas.

Quer que eu monte a linha final bilíngue (Português + Inglês) no formato que costuma aparecer nos artigos — tipo “**Palavras-chave / Keywords:** …” — para você já inserir no documento final conforme o estilo da revista?

Quero ajuda pra começar a sintetizar os principais achados

Perfeito. Agora vamos subir **um nível de abstração**, que é exatamente onde muitos artigos travam — e onde o seu pode ganhar força.

Vou ser **direta e exigente**, como você pediu.

- **1. Antes de escrever: o que não é “sintetizar achados”**

❌ Não é repetir categorias
❌ Não é listar temas (erupção dentária, bruxismo, higiene…)
❌ Não é resumir diálogo por diálogo

Isso já está nos **Resultados**.

👉 **Síntese** é responder:
**“O que tudo isso junto nos mostra sobre educação em saúde, letramento e uso do WhatsApp?”**

- **2. Quais são, de fato, os principais achados do seu estudo?**

A partir do que você trouxe, seus achados centrais **não são clínicos**, são **educacionais e comunicacionais**. Eu sintetizaria em **4 eixos fortes**:

**Eixo 1 — O WhatsApp como espaço ativo de aprendizagem em saúde**

- Mães **não apenas recebem informação**, mas:
  - perguntam
  - validam
  - complementam
  - confrontam
- O aprendizado acontece **no diálogo**, não só na resposta profissional.

👉 Achado:

A educação em saúde ocorre de forma processual, social e situada.

**Eixo 2 — Diferentes níveis de letramento em saúde coexistem no grupo**

Você mostrou claramente:

- mães com autonomia e pensamento crítico
- mães com dificuldade de compreensão/aplicação
- mães que reproduzem informações equivocadas

👉 Achado:

O grupo evidencia a heterogeneidade do letramento em saúde materno, indo do nível funcional ao crítico.

Aqui Nutbeam **encaixa perfeitamente** (sem precisar forçar).

**Eixo 3 — O papel estruturante (e insubstituível) do profissional de saúde**

Não é só “responder dúvidas”.

Você mostrou que o profissional:

- traduz
- corrige
- contextualiza
- previne desinformação
- personaliza o cuidado (mensagem privada, imagens, áudios)

👉 Achado:

A presença profissional qualifica o ambiente digital e transforma interação em educação em saúde.

**Eixo 4 — Potencial educativo × risco de desinformação (infodemia)**

Muito forte no seu material:

- gentian violet
- experiências adultas aplicadas a bebês
- conselhos sem base científica

👉 Achado:

O mesmo ambiente que promove apoio e aprendizado também favorece a circulação de informações não confiáveis, exigindo mediação ativa.

- **3. Como transformar isso em texto de síntese**

Agora, vou te dar **um parágrafo-modelo de síntese dos principais achados**, que normalmente entra logo após o parágrafo introdutório da Discussão ou como subtópico “Principal Findings”.

**Parágrafo de síntese dos achados**

Overall, the findings demonstrate that the WhatsApp group functioned as a relevant digital space for maternal health education, combining information exchange, peer support, and professional guidance. Mothers actively engaged in seeking, sharing, and interpreting information related to child oral health, revealing different levels of health literacy, from basic information seeking to more critical and reflective engagement. The interaction between mothers and health professionals played a key role in transforming doubts into learning opportunities, while also mitigating the circulation of misinformation. At the same time, peer-generated content, although valuable for emotional support and experience sharing, exposed vulnerabilities related to the accuracy of health information. These results highlight both the educational potential and the inherent risks of digital communication tools in the context of parental health education.

Esse parágrafo:

- **não cita diálogos**
- **não entra em temas específicos**
- cria uma **linha mestra** que depois se desdobra nas categorias
- **4. Como seguir daqui (estratégia prática)**

Agora, a Discussão flui assim:

1. **Parágrafo introdutório** (já fizemos)
2. **Parágrafo de síntese dos achados** (acima)
3. Depois, sim:
   - Eruption of Primary Teeth → exemplo de aprendizagem + suporte
   - Medication → letramento funcional vs aplicação
   - Bruxism → mediação profissional
   - Audiovisual → novas formas de comunicação
   - Valuing the group → apoio social

Cada subtema passa a ser **evidência** do que você já sintetizou.
